# Endoscopic and histopathological phenotypes of early gastric neoplasia: toward an integrative host-response framework

**DOI:** 10.3389/fonc.2026.1889434

**Published:** 2026-06-29

**Authors:** Ting Wang, Bin Zhou

**Affiliations:** 1 Guang’anmen Hospital South Campus, China Academy of Chinese Medical Sciences, Beijing, China; 2 Guang’anmen Hospital, China Academy of Chinese Medical Sciences, Beijing, China

**Keywords:** early gastric neoplasia, gastric carcinogenesis, histopathology, host-response phenotype, image-enhanced endoscopy, syndrome differentiation

## Abstract

**Background:**

Early gastric neoplasia represents a clinically decisive but biologically heterogeneous interval in gastric carcinogenesis. Contemporary endoscopy has improved lesion detection and characterization through white-light endoscopy, linked color imaging, blue laser imaging, magnifying narrow-band imaging, and computer-aided systems. Histopathology remains essential for defining dysplasia, invasion depth, differentiation, mucin phenotype, and endoscopic curability. However, most current diagnostic frameworks remain predominantly lesion-centered and incompletely account for the injured mucosal field and host-response context in which early neoplastic lesions arise.

**Objective:**

This review aims to synthesize endoscopic, histopathological, microenvironmental, microbial, metabolic, and integrative medicine evidence to propose a lesion–field–host framework for interpreting early gastric neoplasia.

**Evidence acquisition:**

We reviewed key evidence from endoscopic imaging studies, gastric premalignant lesion guidelines, Helicobacter pylori prevention literature, pathology-continuum studies, metaplasia and SPEM biology, OLGA/OLGIM and Kyoto-classification research, single-cell and spatial profiling, microbiome and metabolomics studies, digital pathology, and syndrome-related clinical research.

**Evidence synthesis:**

The proposed framework comprises three interrelated layers. The lesion layer captures visible and microscopic features of superficial neoplastic disease, including morphology, demarcation line, microvascular and microsurface patterns, biopsy and endoscopic submucosal dissection pathology, differentiation, invasion depth, and curability. The field layer captures background mucosal risk, including H. pylori status, eradication history, atrophy, intestinal metaplasia, SPEM-like metaplastic change, OLGA/OLGIM stage, Kyoto-classification features, microbiome dysbiosis, and metabolomic remodeling. The host-response layer captures inflammatory, immune, metabolic, nutritional, symptom-based, tongue-image, and syndrome-based variables. Within this layer, traditional Chinese medicine syndrome differentiation is framed as a candidate latent host-response phenotype rather than a substitute for endoscopy or histopathology.

**Conclusions:**

Future progress in early gastric neoplasia will likely depend less on isolated biomarkers than on disciplined integration of optical, histological, field-mucosal, and host-response phenotypes. Prospective multicenter validation should incorporate standardized image acquisition, mapping biopsies, OLGA/OLGIM staging, digital pathology, mucosal microbiome and metabolomic profiling, blinded syndrome assessment, and prediction-model reporting aligned with TRIPOD+AI and PROBAST+AI. This framework may support more rational surveillance, prevention, and integrative risk stratification while avoiding overstatement of currently exploratory syndrome–pathology associations.

## 1 Introduction

Gastric cancer remains a major global cancer burden, and its mortality is driven in large part by late-stage presentation. Early gastric cancer and high-grade premalignant lesions are the therapeutic window in which endoscopic treatment can be curative, but the clinical problem is that this window is visually subtle and biologically heterogeneous. A depressed reddish differentiated carcinoma, a flat pale poorly cohesive carcinoma, a lesion partly masked after Helicobacter pylori eradication, and a focus of high-grade dysplasia within extensive intestinal metaplasia are all plausible members of the same clinical problem, yet they do not share a single appearance, molecular context, or background mucosal field (1–4).

The phrase early gastric neoplasia is used here not as a loose synonym for small cancer, but as a practical clinical interval in which optical diagnosis, histological risk, and prevention intersect. This distinction matters for readers outside East Asian screening systems. In high-incidence settings, early lesions are often found during planned surveillance or opportunistic screening; in lower-incidence settings, comparable lesions may be encountered in the work-up of anemia, dyspepsia, Barrett-like symptoms, or follow-up of gastric intestinal metaplasia. The framework must therefore be robust across different pretest probabilities, endoscopic expertise, and biopsy practices rather than assuming an idealized expert ESD environment (2, 4–6). Endoscopy and histopathology have each matured substantially. The endoscopic discipline now includes structured white-light inspection, chromoendoscopy, magnifying narrow-band imaging, blue laser imaging, linked color imaging, and computer-assisted detection or characterization. Histopathology, in parallel, defines the severity of chronic gastritis, activity, atrophy, intestinal metaplasia, dysplasia, mucin phenotype, lymphovascular invasion, and submucosal invasion. Guidelines for early gastric cancer diagnosis and for the management of premalignant gastric conditions have standardized many of these variables (2, 6–9).

However, a gap remains between lesion recognition and biological interpretation. A lesion-centered approach asks whether a visible abnormality is neoplastic, how far it invades, and whether it is endoscopically resectable. Those questions are essential, but they do not fully capture field cancerization, background inflammatory ecology, post-eradication mucosal remodeling, or host-level vulnerability. Contemporary research in single-cell transcriptomics, spatial biology, gastric microbiome science, metabolomics, and computational pathology is increasingly showing that early neoplasia is not only a focal epithelial event but also an expression of a wider lesion-field-host process (10–14). This gap is increasingly relevant because the more successful endoscopy becomes, the more it reveals biological ambiguity. A visible abnormality can be endoscopically suspicious yet biologically indolent, while a deceptively mild field may contain focal high-grade dysplasia. Conversely, a patient can have extensive atrophy and intestinal metaplasia without a visible neoplastic target at a given examination. A useful review should therefore not merely list endoscopic signs and histological categories; it should show how the signs operate within a damaged field and how a host phenotype might help explain persistence, recurrence, metachronous disease, or progression risk.

This review advances a specific argument: early gastric neoplasia should be interpreted simultaneously as an optical phenotype, a histopathologic state, a field-mucosal process, and a host-response construct. The argument is not that host-response features can replace endoscopy or pathology. Rather, it is that endoscopic and histological phenotypes become more clinically informative when interpreted together with the mucosal field and host state in which they arise. Within that host-response layer, traditional Chinese medicine syndrome differentiation is discussed as a candidate latent phenotyping system, not as a cancer diagnostic system. This distinction is central. Current evidence does not justify assigning a specific endoscopic microvascular pattern or histological subtype to a fixed syndrome label. It does justify asking whether standardized syndrome information, collected independently and blindly, can contribute to risk stratification when analyzed alongside endoscopy, histology, microbiome, metabolomic, and inflammatory variables (15–20).

The language of host response also prevents a common category error in integrative oncology writing. A symptom pattern, a tongue-coating feature, or a syndrome label is not equivalent to a molecular pathway. It is a composite observation that may reflect several biological, behavioral, and constitutional variables. Such constructs become scientifically useful only when they are collected before outcome disclosure, translated into measurable variables, and tested against conventional predictors. This is why the review treats syndrome differentiation as a latent clinical phenotype rather than as proof of a distinct cancer biology.

The purpose of this review is therefore to synthesize the evidence in a way that produces a testable research framework. We first define early gastric neoplasia and its lesion-field-host architecture. We then discuss endoscopic phenotypes, histopathological and molecular states, microenvironmental remodeling, image-pathology correlations, and syndrome-based host-response phenotyping. Finally, we propose methodological standards for prospective multicenter studies capable of testing whether integrative phenotyping improves surveillance, early detection, and individualized prevention.

## 2 Literature identification and evidence appraisal

This is a narrative, state-of-the-art review rather than a systematic review or meta-analysis. A PubMed-focused literature search was performed through 18 May 2026, prioritizing guideline documents, prospective diagnostic studies, multicenter cohorts, meta-analyses, mechanistic pathology studies, single-cell or spatial studies, microbiome and metabolomic studies, digital pathology and AI methodology papers, and PubMed-indexed studies on syndrome differentiation or tongue-image phenotyping in gastric disease. Search concepts included early gastric cancer, early gastric neoplasia, gastric dysplasia, gastric intraepithelial neoplasia, magnifying endoscopy, narrow-band imaging, blue laser imaging, linked color imaging, Kyoto classification, OLGA, OLGIM, intestinal metaplasia, SPEM, paligenosis, Helicobacter pylori eradication, gastric microbiome, gastric metabolomics, single-cell gastric premalignant lesions, digital pathology gastric cancer, TCM syndrome gastric cancer, tongue diagnosis gastric cancer, and machine learning risk prediction.

Studies were not treated as equivalent merely because they addressed gastric cancer. A prospective multicenter ME-NBI study carries different evidentiary weight for optical diagnosis than a single-center retrospective image study; an OLGA/OLGIM follow-up cohort carries different weight for field risk than a cross-sectional symptom survey; and a tongue-image AI paper carries different weight for host-screening feasibility than for cancer causality. Throughout the review, established standards are separated from emerging evidence and from explicit hypotheses.

Evidence was weighted according to the question being addressed. For diagnostic endoscopy, prospective and multicenter studies, diagnostic meta-analyses, and guideline statements were prioritized (2, 21–23). For premalignant disease management, MAPS II/MAPS III, OLGA/OLGIM evidence, and Kyoto-classification literature were prioritized (6, 9, 24–26). For mechanisms, the review considered both classical carcinogenesis concepts and newer lineage-state, microenvironmental, microbial, and spatial data (10, 12, 27–30). For syndrome-related evidence, the appraisal was deliberately conservative because studies are heterogeneous in diagnostic criteria, stage distribution, blinding, endpoints, and reproducibility (16, 20, 31, 32).

The expanded reference set was deliberately broadened beyond the initial core bibliography. It now includes seminal carcinogenesis papers, modern MAPS guidance, OLGA/OLGIM evidence, ME-NBI and LCI/BLI studies, post-eradication work, H. pylori prevention evidence, single-cell and spatial studies, microbiome and metabolomic data, computational pathology, reporting standards for prediction models, and PubMed-indexed TCM or digital tongue-phenotyping studies. This breadth is necessary because the manuscript’s proposed contribution is integrative; a narrow bibliography would make the framework appear rhetorical rather than evidence-grounded.

## 3 Defining early gastric neoplasia as a lesion-field-host process

The term early gastric neoplasia is useful only if its boundaries are explicit. In this review, it refers to a clinically relevant continuum that includes gastric dysplasia or intraepithelial neoplasia, high-grade dysplasia, intramucosal carcinoma, and superficial submucosal carcinoma when discussed in relation to early detection and endoscopic management. Intestinal metaplasia and SPEM-like metaplastic states are not themselves neoplasia, but they are included because they form the field in which neoplasia is detected, sampled, interpreted, and surveilled (27, 28, 33–35).

Premalignant conditions are included for two reasons. First, early cancer is usually detected within an already altered mucosal field, and sampling or surveillance decisions often depend on the severity and distribution of that field. Second, the most interesting host-response question may not be whether a syndrome label correlates with an already resected cancer, but whether it correlates with the field severity or biological ecology that precedes visible neoplasia. This is why atrophy, complete versus incomplete intestinal metaplasia, and SPEM-like metaplastic states are treated as field phenotypes rather than as peripheral background (34, 36–38). The lesion phenotype is the most familiar layer. It includes the size, location, Paris morphology, color, surface depression or elevation, ulceration, demarcation line, microvascular pattern, microsurface pattern, histological differentiation, invasion depth, mucin phenotype, and lymphovascular status of the focal abnormality. Lesion phenotyping informs whether a target is suspicious, whether biopsy or endoscopic resection is indicated, whether ESD is curative, and whether the patient needs surgery or closer follow-up (3, 4, 39, 40).

**Figure 1:**
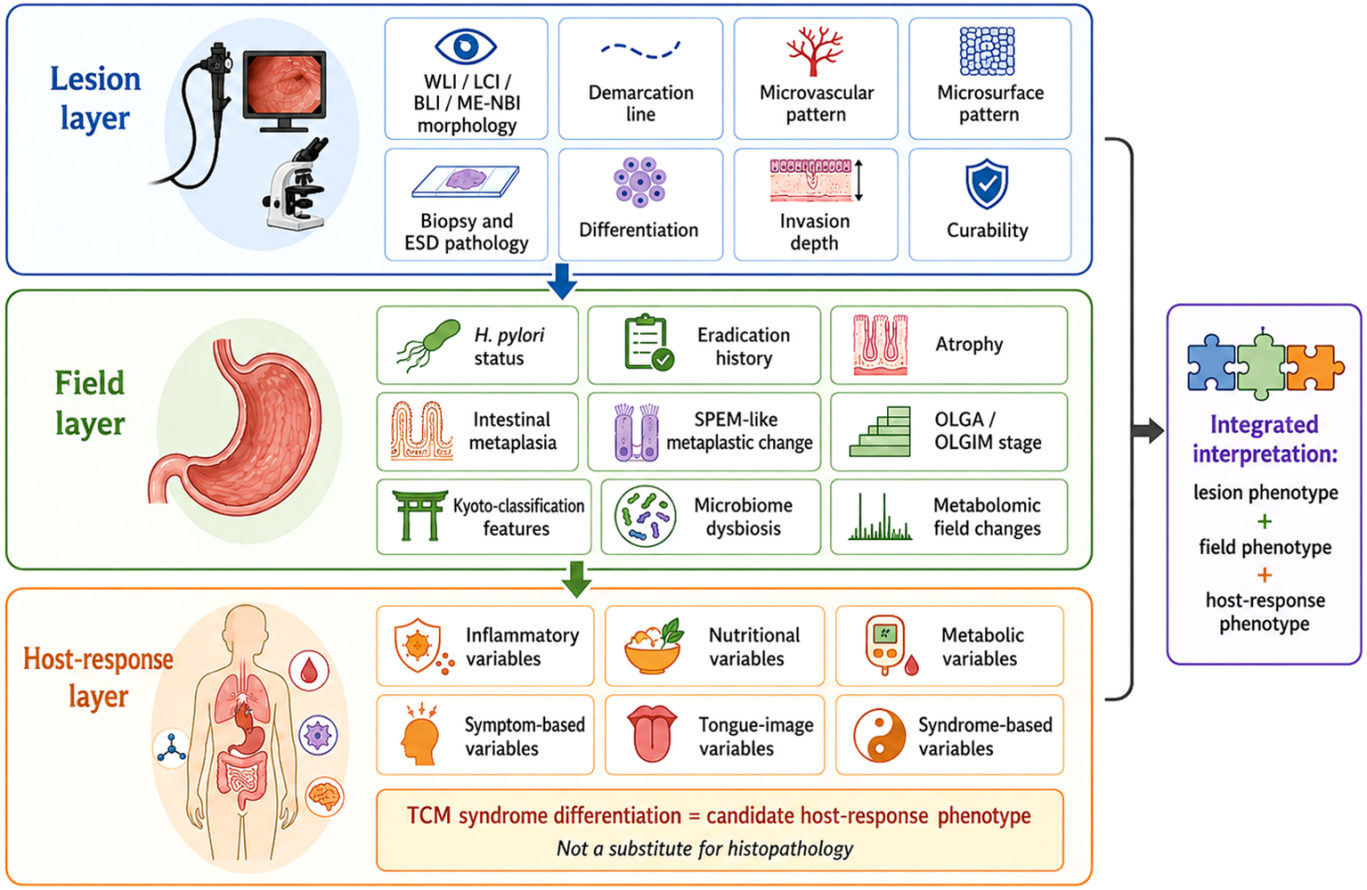
Lesion–field–host framework for early gastric neoplasia. The lesion layer is anchored to endoscopic detection, biopsy, and resection pathology; the field layer reflects longer-term mucosal injury and carcinogenic memory; and the host-response layer captures time-stamped inflammatory, nutritional, metabolic, symptom-based, tongue-image, and syndrome-based variables. Traditional Chinese medicine syndrome differentiation is positioned as a candidate dynamic host-response state, not as a substitute for histopathology.

The field phenotype refers to the background gastric mucosa. This layer includes H. pylori infection or eradication history, chronic active inflammation, glandular atrophy, distribution and subtype of intestinal metaplasia, OLGIM/OLGA stage, endoscopic Kyoto-classification features, map-like redness after eradication, and microbial or metabolomic perturbations. Field phenotyping is essential because the same lesion morphology has a different clinical meaning when arising in a stomach with diffuse incomplete intestinal metaplasia, severe corpus atrophy, or persistent dysbiosis after eradication (6, 8, 9, 26, 41, 42). The host-response phenotype is the least standardized but perhaps the most neglected. It includes host inflammatory tone, immune-cell ecology, nutritional and metabolic state, symptoms, performance status, mucosal barrier function, and observable clinical features that may not be captured by routine histology. Traditional Chinese medicine syndrome differentiation can be placed here, provided it is treated as a composite clinical phenotype rather than as a substitute for disease classification. The value of this three-level model is that it discourages a false dichotomy between focal lesion diagnosis and systemic interpretation. Early neoplasia is visible at the lesion layer, evolves within the field layer, and is conditioned by the host-response layer. This three-layer architecture is summarized in **Figure 1**.

A host-response phenotype is not synonymous with TCM. It can include C-reactive protein, albumin, sarcopenia, cytokines, plasma metabolites, symptom burden, medication exposure, mucosal barrier status, and microbiome-related signals. Syndrome differentiation is only one candidate representation of this layer. Placing it inside a broader host-response construct gives it a defensible translational role while protecting the manuscript from the unsupported claim that traditional syndromes are disease entities equivalent to histological diagnoses.

## 4 Endoscopic phenotypes: detection, characterization, background risk, and post-eradication change

White-light endoscopy remains the entry point for early detection. Its strengths are availability, panoramic assessment, documentation of lesion morphology, and recognition of obvious color or surface change. Its weakness is that early neoplastic lesions often mimic gastritis, erosion, scar, or intestinal metaplasia. Flat and depressed lesions, small undifferentiated lesions, and lesions after H. pylori eradication are particularly likely to be missed if inspection time, mucosal cleansing, photodocumentation, and targeted re-examination are inadequate (2, 43, 44). Detection is procedural as much as optical. Mucosal preparation, antifoaming agents, complete photodocumentation, inspection time, retroflexion, adequate distension, training, and systematic reinspection all influence whether a small lesion is even available for characterization. This is why computer-aided quality control and risk-scoring systems may eventually matter as much as lesion-classification algorithms. In a lesion-field-host framework, poor detection quality creates missing lesion data and can falsely shift attention to field or host variables.

Magnifying endoscopy with narrow-band imaging has contributed the most durable diagnostic vocabulary for lesion characterization. The vessel-plus-surface framework emphasizes the presence of a demarcation line and irregular microvascular or microsurface patterns within that line. Prospective studies and meta-analyses have consistently shown that ME-NBI improves qualitative diagnosis compared with white-light inspection, particularly for lesions already detected as suspicious (21–23, 45, 46). The magnifying endoscopic simple diagnostic algorithm for early gastric cancer translated these concepts into a practical clinical sequence and helped separate diagnostic characterization from simple visual detection (40).

The diagnostic evidence for ME-NBI should also be read historically. Early work established that narrow-band imaging could highlight microvascular architecture; later prospective studies and comparative analyses demonstrated improved accuracy for superficial gastric lesions and early gastric cancer; subsequent algorithms simplified the decision process for clinical use (21, 40, 45–48). This staged development explains why ME-NBI is treated in this review as the strongest lesion-characterization modality rather than simply as another image-enhancement option.

Yet ME-NBI should not be overinterpreted. Optical microvascular and microsurface patterns do not directly equal histological type, and the performance of ME-NBI depends on lesion type, operator experience, the quality of magnification, and the biological characteristics of the lesion. Some undifferentiated cancers, poorly cohesive carcinomas, and post-eradication lesions may have subdued color, partially restored surface epithelium, or less conspicuous demarcation. The framework proposed here therefore treats ME-NBI as a high-resolution lesion-phenotyping tool, not as a complete biological classifier (21, 44, 49). Blue laser imaging and linked color imaging are complementary rather than redundant. BLI can enhance vascular and surface patterns and has been evaluated for the diagnosis of early gastric cancer; LCI improves color contrast and may increase visibility of subtle neoplastic or background mucosal changes, including intestinal metaplasia and post-eradication patterns. Randomized and observational studies suggest that LCI may improve detection in high-risk populations, whereas ME-NBI and BLI are more often used for detailed characterization of a suspicious focus (50–55).

LCI deserves particular nuance. Its contribution is often visibility rather than definitive diagnosis: it may make a lesion stand out against intestinal metaplasia, inflammation, or post-eradication background, but it usually does not remove the need for ME-NBI characterization or histological confirmation. Earlier LCI studies emphasized color contrast and improved recognition, and later randomized or AI-assisted work extended the argument to detection performance and quantitative support (51–53, 56, 57). This distinction helps avoid overstating LCI as a complete diagnostic replacement for magnified inspection.

Endoscopic assessment of background mucosa deserves separate treatment because it is not the same as lesion diagnosis. Kyoto-classification features such as atrophy, intestinal metaplasia, enlarged folds, nodularity, diffuse redness, and map-like redness provide information about H. pylori status and gastric cancer risk. These findings are better understood as field phenotypes. They should be documented even when the focal lesion appears small, because background atrophy and metaplasia influence metachronous cancer risk and surveillance planning (6, 9, 26).

Post-eradication neoplasia is a particularly important stress test for the lesion-field-host framework. H. pylori eradication reduces gastric cancer risk, including metachronous cancer risk after endoscopic resection, but does not erase the accumulated field injury in patients with advanced atrophy or intestinal metaplasia. After eradication, cancers may present as flatter, less inflamed, or epithelium-covered lesions, and the background mucosa may show map-like redness or altered microbial composition. Such lesions illustrate why early detection must combine lesion scrutiny with field memory and eradication history (42, 44, 58–62).

The post-eradication stomach also illustrates why time must be recorded as a variable. Risk after eradication depends on age, baseline atrophy, intestinal metaplasia, prior neoplasia, interval from eradication, and quality of follow-up. A binary variable of ever-eradicated versus never-eradicated is therefore insufficient. In future studies, eradication history should be linked to background mucosal photographs, OLGA/OLGIM stage where available, and metachronous outcome. This approach is more clinically meaningful than asking whether eradication simply normalizes the stomach (58, 60, 61). Artificial intelligence is beginning to shift endoscopy from visual interpretation to quantitative phenotype extraction. CNN-based systems have shown promise for gastric cancer detection, quality control of upper gastrointestinal endoscopy, real-time characterization under image-enhanced endoscopy, margin delineation, differentiation-status estimation, and background risk prediction (51, 63–67). The risk, however, is that AI models trained on selected images may reproduce spectrum bias, endoscopist-selection bias, and center-specific acquisition patterns. For this review, the relevant contribution of AI is not the assertion that algorithms can replace endoscopists, but the possibility that optical phenotypes can be quantified and linked to pathology, field features, and host-response variables under externally validated protocols.

AI studies also make clear that the unit of analysis must be specified. Image-level performance can look excellent while patient-level or examination-level performance is less certain. A still image chosen by an expert endoscopist is not the same as a full screening examination with bubbles, blind spots, rapid scope movement, variable insufflation, and incomplete views. Therefore, AI in this field should be developed with external validation at image, lesion, patient, and center levels, and should be benchmarked against human endoscopists working under realistic clinical constraints (63–66). The principal endoscopic phenotypes, interpretive contributions, and practical limitations of each modality are summarized in **Table 1**.

**Table 1:** Endoscopic phenotypes of early gastric neoplasia and their interpretive role.

Domain	Principal variables	What it contributes	Key limitations
White-light imaging	Color change, depression/elevation, erosion, ulcer scar, fold change, Paris morphology, location, size	First-line detection and gross morphological documentation; essential for screening and lesion mapping	Limited sensitivity for flat, pale, minute, post-eradication, and poorly cohesive lesions; quality depends on inspection time and preparation
ME-NBI	Demarcation line, irregular microvascular pattern, irregular microsurface pattern, vessel-plus-surface classification, MESDA-G logic	High-resolution characterization of detected lesions; improves distinction between neoplastic and non-neoplastic mucosa	Not a substitute for histology; reduced confidence in some undifferentiated or post-eradication lesions; operator-dependent
BLI	Enhanced vascular and surface contrast under laser-based illumination	Supports detailed lesion characterization and may complement ME-NBI in selected settings	Evidence base smaller than NBI; modality availability varies
LCI	Color contrast, visibility against intestinal metaplasia or inflamed mucosa, background mucosal patterns	Detection-oriented modality; useful for subtle lesions and background field assessment	May improve visibility without always resolving histological category; requires standardized acquisition
Background mucosal phenotyping	Kyoto features, atrophy, intestinal metaplasia, map-like redness, nodularity, enlarged folds, diffuse redness	Assesses field risk and H. pylori or post-eradication context	Should not be confused with lesion-specific diagnosis; interobserver variability remains relevant
AI-assisted endoscopy	CADe, CADx, quality control, risk prediction, margin delineation, differentiation estimation	Enables quantitative optical phenotyping and standardization; can support detection and characterization tasks	Spectrum bias, external validity, calibration, and workflow integration remain major barriers

## 5 Histopathological phenotypes and the biological continuum

Histopathology remains the reference standard because it resolves biological states that endoscopy can only infer. The updated Sydney System formalized the grading of chronic inflammation, activity, atrophy, intestinal metaplasia, and H. pylori density. OLGA and OLGIM systems converted those histological findings into staged cancer-risk frameworks, and long-term follow-up and meta-analytic evidence support their role in risk stratification (7, 8, 24, 25, 68). MAPS II and MAPS III place these staging concepts into surveillance and management recommendations for premalignant gastric conditions and early neoplasia (6, 9).

Sampling strategy is a major determinant of field interpretation. Targeted biopsies can diagnose a visible lesion but may underestimate the distribution of atrophy or metaplasia; random mapping biopsies can stage the field but may miss focal dysplasia; ESD specimens provide excellent lesion-level tissue but only a partial window into the surrounding field. An integrative study should therefore predefine whether pathology is being used to classify a lesion, stage a background field, or validate a multimodal risk model. Mixing these roles without distinction produces weak conclusions.

The classical Correa cascade remains the best-known model for intestinal-type gastric carcinogenesis: chronic gastritis, multifocal atrophic gastritis, intestinal metaplasia, dysplasia, and carcinoma. Its strength is clinical utility: it provides an ordered language for biopsy interpretation and surveillance. Its limitation is that it can be misread as deterministic or exclusive. In practice, the mucosa may contain mixed tissue states, incomplete regression after eradication, multiple clones, and divergent pathways, particularly in diffuse-type or poorly cohesive disease (27, 28, 33, 69, 70).

A modern pathology section should also recognize that metaplasia is a repair-associated state as much as a premalignant label. Work on paligenosis and injury-induced reprogramming suggests that mature epithelial cells can re-enter proliferative and regenerative programs under stress, while ADAR1, SOX9, DDIT4, mTORC1, and other pathways have been implicated in the regulation or selection of metaplastic states (29, 71–75). This literature strengthens the rationale for treating the mucosal field as a dynamic biological compartment rather than as a static histological label. Intestinal metaplasia itself is not a uniform field. Complete and incomplete subtypes differ in morphology, mucin expression, and risk implication; incomplete intestinal metaplasia is often considered a higher-risk phenotype. Molecular studies are also beginning to map spatial genomic and transcriptomic heterogeneity within metaplastic mucosa, which suggests that the field can be biologically heterogeneous even when it appears histologically similar at low magnification (13, 34, 36, 76, 77).

**Figure 2:**
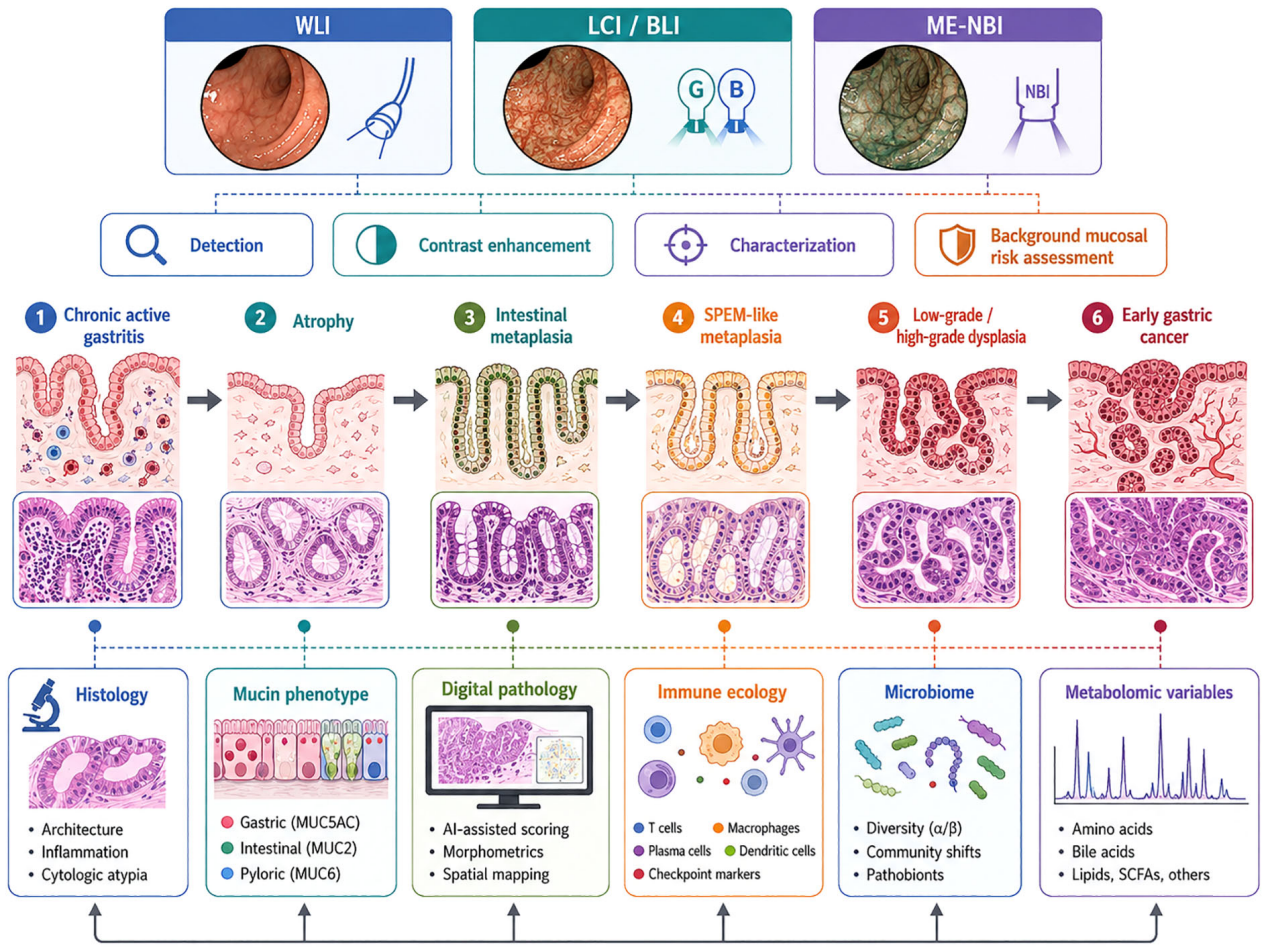
Endoscopic-pathological continuum from chronic injury to early gastric neoplasia. The figure should depict chronic active gastritis, atrophy, intestinal metaplasia, SPEM-like metaplasia, low-grade/high-grade dysplasia, and early gastric cancer. Above the sequence, illustrate WLI, LCI/BLI, and ME-NBI tasks: detection, contrast enhancement, characterization, and background mucosal risk assessment. Below the sequence, depict histology, mucin phenotype, digital pathology, immune ecology, microbiome, and metabolomic variables.

SPEM and paligenosis have added depth to the old cascade. Spasmolytic polypeptide-expressing metaplasia is associated with oxyntic atrophy and chronic injury, and experimental lineage studies suggest that chief-cell reprogramming, mucous neck cell programs, and injury-induced metaplastic regeneration can create transitional epithelial states. Paligenosis provides a conceptual model for how differentiated cells re-enter a reparative proliferative program under injury. These ideas do not replace the clinical importance of intestinal metaplasia, but they broaden the field phenotype beyond an intestinal-only narrative (29, 30, 35, 37, 38, 75, 78, 79). **Figure 2** illustrates this endoscopic–pathological continuum, aligning WLI, LCI/BLI, and ME-NBI tasks with histological and biological annotation layers from chronic active gastritis to early gastric cancer.

For early cancer, histological type and invasion depth drive management. Differentiated intramucosal lesions without lymphovascular invasion are often amenable to endoscopic submucosal dissection when guideline criteria are satisfied, whereas undifferentiated histology, ulceration, deep submucosal invasion, lymphovascular invasion, and positive margins alter curability assessment and follow-up. Endoscopic appearance may suggest some of these features, but histology is needed to adjudicate them (2–4). ESD specimens are underused in translational phenotype research. They contain the lesion, its lateral and vertical architecture, the surface epithelium, the invasive front, and often adjacent non-neoplastic mucosa. If paired with pre-resection WLI/LCI/BLI/ME-NBI images and digitized whole-slide pathology, ESD material could become a powerful resource for mapping optical signs to histology, mucin phenotype, immune infiltration, and molecular features. This is a practical opportunity because ESD is already performed as standard care in many early gastric cancer settings. **Table 2** summarizes the major pathological and molecular correlates that anchor lesion–field interpretation and provide candidate measurements for future integrative studies.

**Table 2:** Pathological and molecular correlates relevant to lesion-field interpretation.

Pathological or molecular phenotype	Field or lesion layer	Clinical relevance	Candidate measurements
Chronic active gastritis	Field	Indicates ongoing inflammatory pressure and H. pylori-related activity	Updated Sydney activity/inflammation; H. pylori testing; cytokine markers
Atrophy	Field	Represents loss of glands and risk-field maturation	Sydney grading; OLGA stage; endoscopic atrophic border
Complete/incomplete intestinal metaplasia	Field	Risk varies by distribution and subtype; incomplete IM often higher-risk	Histochemical or mucin markers; OLGIM stage; mapping biopsies
SPEM-like metaplasia and paligenosis	Field/transition state	Represents injury-repair reprogramming beyond an intestinal-only pathway	TFF2/SPEM markers, AQP5, lineage markers, spatial transcriptomics
Dysplasia/HGD	Lesion within field	Defines high-risk neoplastic transformation and management urgency	Histological grade; Ki-67; p53; digital pathology
Early gastric cancer	Lesion	Determines ESD/surgery decision and surveillance	Differentiation, invasion depth, ulceration, margins, lymphovascular invasion
Mucin phenotype	Lesion/lineage	Bridges optical phenotype with epithelial differentiation	MUC2, MUC5AC, MUC6, CDX2, mucin phenotype classification
Microvascular remodeling	Lesion and microenvironment	May underlie irregular MV patterns and angiogenic risk	ME-NBI/BLI scoring; CD31/CD34; VEGF; multiplex IHC
Immune-stromal ecotype	Field/host	May explain progression-prone mucosal neighborhoods	Single-cell RNA-seq; spatial profiling; multiplex IHC; digital pathology
Microbiome/metabolome	Field/host	Captures microbial and biochemical context not visible by routine histology	Mucosal microbiome; plasma/tissue metabolomics; eradication history

The important interpretive point is that endoscopic and histological phenotypes are coupled but not interchangeable. Irregular vessels may reflect angiogenic remodeling, glandular crowding, or surface destruction; a white opaque substance may reflect lipid droplet accumulation; a white globe appearance may mark intraglandular necrotic material, yet these signs are probabilistic optical markers rather than definitive biological categories. A high-quality review must therefore emphasize the bridge between optics and tissue, while preserving the authority of histological diagnosis.

## 6 Microenvironmental ecology across the gastric precancerous-to-neoplastic sequence

The microenvironment should be discussed as an evolving ecology rather than as a static list of cytokines. H. pylori is a major initiating and promoting factor in non-cardia gastric carcinogenesis, and the association is supported by epidemiology, mechanistic studies, and prevention trials. The bacterium induces epithelial injury, inflammatory-cell recruitment, oxidative stress, immune modulation, and altered gastric acidity; eradication reduces risk, particularly before irreversible field injury has accumulated (60, 80–85). Prevention trials further illustrate the field nature of disease. H. pylori eradication can reduce risk, but the magnitude of benefit is conditioned by timing and baseline mucosal damage. In earlier stages, eradication may interrupt inflammatory promotion; in advanced atrophy or intestinal metaplasia, it may reduce but not eliminate risk. This is one reason why guidelines continue to emphasize surveillance of extensive atrophy or intestinal metaplasia after eradication rather than assuming that infection cure fully resets the field (6, 9, 58, 59, 83).

Inflammation is the first host-response axis. Chronic active gastritis recruits neutrophils, macrophages, lymphocytes, and stromal cells, with downstream activation of inflammatory and epithelial stress pathways. Cytokines such as IL-6 and TNF-alpha, NF-kappaB signaling, COX-2 induction, and persistent epithelial turnover provide plausible biological bridges from infection to metaplasia and dysplasia. The key point is that the inflammatory field is not merely background noise; it modifies epithelial repair and selection pressure (32, 86, 87). Barrier dysfunction adds another dimension, because epithelial tight-junction damage and increased permeability can amplify microbe-host crosstalk across the Correa cascade (88). Immune remodeling becomes progressively more complex as tissue states evolve. Single-cell studies across premalignant and malignant lesions have identified lineage diversity, epithelial-state transitions, immune-cell heterogeneity, stromal remodeling, and ecotypes associated with disease progression. These data move the field beyond the simple question of whether immune cells are present. They raise a more precise question: which epithelial, immune, and stromal neighborhoods emerge before invasive disease, and do they correspond to optical, pathological, or host-response phenotypes? (10–12, 89, 90).

This immune-stromal perspective gives the host-response layer biological plausibility. A syndrome or symptom cluster might correlate poorly with a focal lesion but more meaningfully with inflammatory activity, stromal remodeling, or systemic metabolic response. That possibility should be tested, not assumed. The question becomes whether host-observable variables identify a reproducible ecological state in the mucosa, such as inflammation-dominant, metaplasia-dominant, immune-excluded, macrophage-enriched, or metabolically depleted fields (10, 12, 89, 91). The gastric microbiome is not reducible to H. pylori, even though H. pylori remains the dominant carcinogenic organism. Studies of gastric microbiota in intestinal metaplasia, post-eradication stomachs, and gastric cancer suggest stage-dependent dysbiosis, altered functional pathways, and long-term microbial perturbation in some high-risk settings. The field must avoid simplistic claims that any single non-H. pylori microbial signature causes early gastric cancer, but microbiome data are a strong candidate field-host layer for integrative phenotyping (14, 41, 42, 92–96).

Microbiome studies also require careful interpretation. Sampling site, biopsy technique, proton-pump inhibitor use, antibiotics, eradication therapy, sequencing platform, contamination control, and gastric acidity can all alter results. A microbial signature that appears associated with gastric cancer in one study may reflect field severity, medication, or geography rather than causal biology. Future lesion-field-host studies should therefore record technical and clinical covariates and should avoid treating dysbiosis as a single universal biomarker (14, 92, 93, 96). Metabolism provides a complementary host-state axis. Metabolomic analyses across the Correa cascade, plasma biomarker studies in high-risk populations, and comparisons between intestinal metaplasia and gastric cancer suggest that biochemical shifts accompany histological progression. These shifts may reflect epithelial reprogramming, microbial metabolism, inflammation, bile acid exposure, hypoxia, oxidative stress, and nutritional status. Metabolomics is particularly relevant to the host-response framework because it can capture systemic signals not visible on routine endoscopy (97–100).

**Figure 3:**
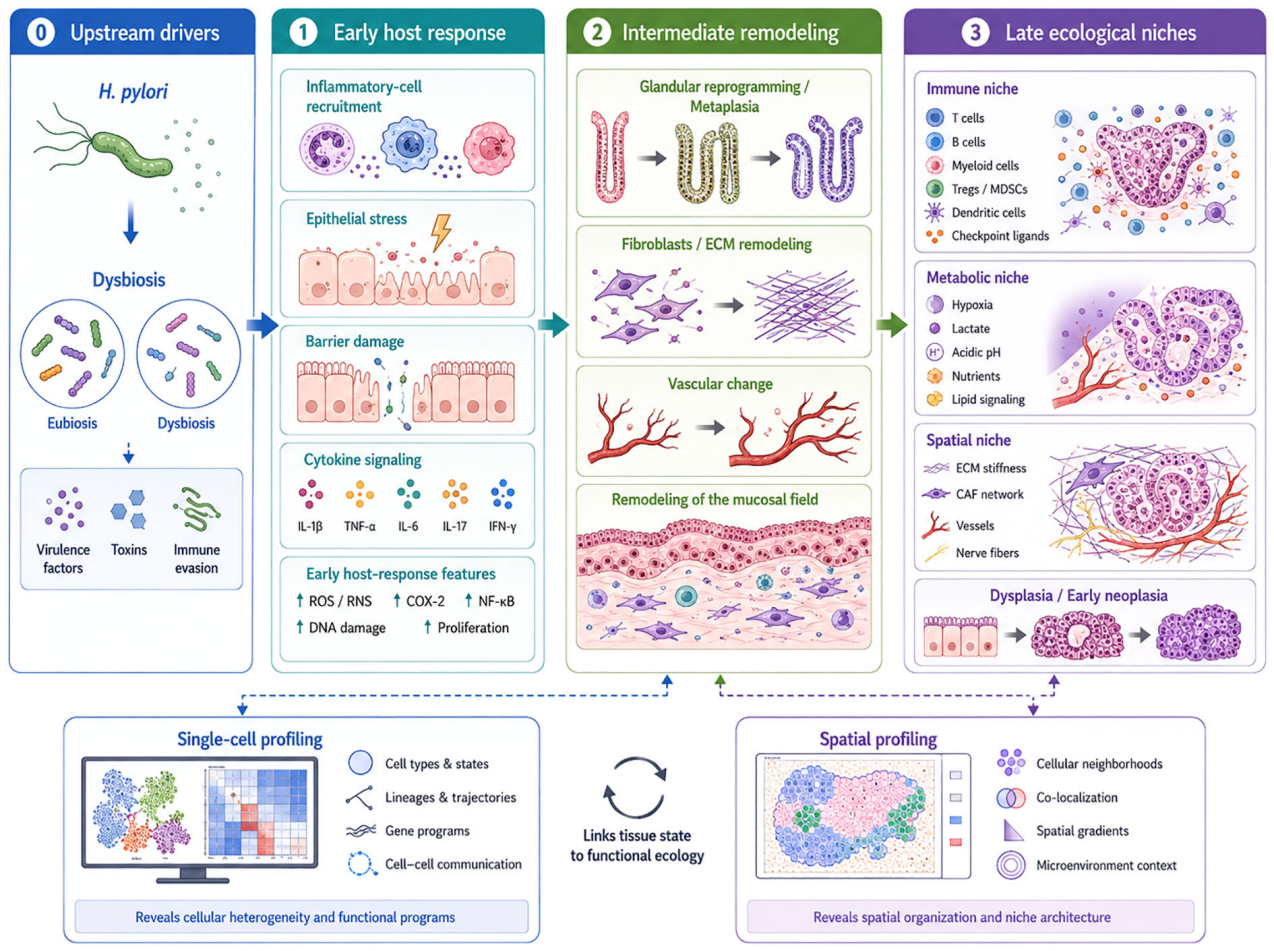
Microenvironmental evolution across gastric carcinogenesis. The figure should show H. pylori and dysbiosis as upstream drivers; inflammatory-cell recruitment and epithelial stress as early host-response states; metaplastic epithelial reprogramming and stromal remodeling as intermediate states; and immune, metabolic, and spatial niches supporting dysplasia and early neoplasia. Single-cell and spatial profiling are shown as technologies that connect tissue state to functional ecology.

Spatial profiling and multiplex imaging are beginning to close the gap between histology and function. Macrophage spatial heterogeneity, spatially mapped intestinal metaplasia gene expression, and digital whole-slide approaches suggest that tissue neighborhoods can be quantified. This is precisely the level at which the lesion-field-host framework can become testable: optical patterns, biopsy maps, digital histology, immune neighborhoods, microbiome states, metabolomic signatures, and host-observable phenotypes can be analyzed in the same patient rather than reviewed as separate literatures (13, 77, 91, 101). **Figure 3** integrates these observations into a temporal model of microenvironmental evolution, from upstream H. pylori–associated dysbiosis and epithelial stress to immune, metabolic, and spatial niches that support dysplasia and early neoplasia.

## 7 Image-pathology-molecular bridges

A central weakness of many reviews on early gastric neoplasia is that endoscopy and pathology are described in parallel rather than integrated. The integration should begin with established optical-pathologic correlations. ME-NBI microvascular and microsurface patterns reflect, at least in part, abnormal glandular architecture, angiogenic remodeling, epithelial proliferation, and surface maturation. Studies correlating ME-NBI with histopathology and mucin phenotype show that optical structures can carry histological information, but the correlations are not perfect enough to substitute for tissue diagnosis (49, 102). A useful bridge has to be bidirectional. Endoscopic findings can suggest histology, but histology can also explain why an endoscopic sign is unreliable. For example, a surface covered by non-neoplastic epithelium after eradication may blunt the visible cancer signal; intestinal metaplasia can create color contrast that improves or confuses LCI detection; and mucin phenotype can alter surface texture without necessarily changing invasion depth. This is the reason the present review argues for paired image-pathology datasets rather than retrospective narrative linkage.

Mucin phenotype is especially useful for bridging disciplines. Gastric, intestinal, mixed, and null mucin phenotypes influence surface architecture, differentiation, and potentially biological behavior. Endoscopically, these phenotypes may affect whether a lesion shows preserved pits, white opaque substance, or a particular microsurface arrangement. Histologically, they situate the lesion within broader epithelial-lineage programs. Molecularly, they connect to CDX2, MUC2, MUC5AC, MUC6, and related lineage markers. This makes mucin phenotype a practical candidate variable in future lesion-field-host cohorts (49, 70, 102). The white globe appearance and white opaque substance illustrate both the promise and the danger of optical biomarkers. The white globe appearance has been proposed as a marker for early gastric cancer, while interobserver and differential-diagnosis studies have examined its reproducibility and limits. White opaque substance has been linked to lipid droplet accumulation and lipid-metabolism gene expression in gastric neoplasms. These examples show that an endoscopic sign can be biologically meaningful, but they also show why each sign requires validation against histology and mechanistic data rather than being elevated into a deterministic diagnostic rule (103–106).

Computational pathology extends this bridge. Digital whole-slide images can now be mined for features related to prognosis, lymphovascular invasion, treatment responsiveness, immune context, and multicancer tissue patterns. Although many computational pathology studies are not restricted to early gastric neoplasia, they demonstrate that routine hematoxylin-eosin morphology contains extractable information beyond human categorical labels. For this manuscript, the implication is that host-response phenotyping should not be limited to subjective symptom or syndrome labels. It can be coupled with quantitative digital morphology and externally validated machine learning (101, 107–109).

The same logic applies to radiopathomics and multimodal pathology models. Their immediate relevance to early gastric neoplasia is not that they will replace the pathologist, but that they demonstrate the feasibility of extracting latent morphology from routine images and linking it to prognosis, immune context, or treatment response (101, 107–109). If such methods are brought upstream to dysplasia and early cancer, they may provide an objective bridge between qualitative histology and host-response phenotypes.

A future optical-pathologic-molecular bridge should therefore include at least four variable classes: standardized endoscopic image features, structured histology including OLGA/OLGIM and mucin markers, digital pathology features from whole-slide images, and field-host biomarkers such as microbiome, inflammatory, and metabolomic profiles. Only with this level of integration can one ask whether a host-response category adds biological signal beyond conventional risk variables.

## 8 Syndrome differentiation as candidate host-response phenotyping

Syndrome differentiation should be introduced to oncology readers with precision. In this review, traditional Chinese medicine syndrome differentiation is not presented as a means to diagnose early gastric cancer, rule out cancer, define invasion depth, or determine endoscopic resectability. Those functions belong to endoscopy, biopsy, ESD pathology, and guideline-based oncology. Syndrome differentiation is instead considered as a candidate host-response phenotyping system: a structured way of describing symptom clusters, constitutional vulnerability, observable mucosal or body-surface features, and illness behavior in a patient with a defined gastric field or lesion.

In this framework, syndrome differentiation is best conceptualized as a time-stamped host-response state rather than as a fixed trait or a stable disease subtype. Some syndrome-related features may reflect relatively durable host vulnerabilities, such as constitutional tendency, long-term nutritional status, recurrent digestive symptoms, or chronic inflammatory susceptibility. Others may be more dynamic and may change with current inflammatory activity, treatment exposure, mucosal injury, psychological stress, nutritional decline, or cancer-related symptom burden. Therefore, syndrome differentiation should be recorded as a temporally anchored clinical state, ideally before disclosure of endoscopic and pathological findings, and repeated assessment should be considered in longitudinal studies. This interpretation preserves the potential value of syndrome-based phenotyping while avoiding the unsupported assumption that a syndrome label represents a permanent biological subtype of early gastric neoplasia.

This positioning also makes the manuscript more acceptable to an international oncology readership. TCM terminology can appear opaque if presented as an alternative nosology for cancer. It becomes more interpretable when translated into clinical phenotyping language: fatigue, appetite, pain quality, bowel pattern, tongue body, tongue coating, pulse, and constitutional or inflammatory descriptors. The review should therefore keep the original syndrome names for scholarly accuracy but consistently pair them with English operational definitions and measurable correlates.

The available gastric cancer syndrome literature is clinically interesting but methodologically uneven. A survey of 767 gastric cancer patients described syndrome characteristics and remains one of the larger classic datasets in this area. Other studies explored associations between syndrome types and E-cadherin/ICAM-1, VEGF, CDH1 polymorphism, lifestyle factors, and clinical variables. These studies suggest that syndrome categories may capture biological or clinical heterogeneity, but they do not prove causation, do not establish lesion-specific diagnostic value, and often include advanced-stage disease rather than early neoplasia (15–17).

The strongest critique of the syndrome literature is not that it is irrelevant; it is that much of it was not designed to answer the questions now being asked. Stage mixture, treatment exposure, lack of blinded assessment, small subgroups, and variable syndrome taxonomies make it difficult to distinguish cancer biology from symptom burden, chemotherapy effects, or nutritional decline. The revised manuscript therefore uses this literature to justify a research question, not to claim an established diagnostic association (15–17, 32).

In gastric precancerous lesions and chronic atrophic gastritis, the TCM literature is more treatment-oriented than phenotype-oriented. Meta-analyses and reviews have evaluated traditional Chinese medicine for gastric precancerous lesions, chronic atrophic gastritis, or lesion transformation, but trial heterogeneity, variable formula composition, inconsistent endpoints, and incomplete blinding limit direct translation to early-neoplasia phenotyping (31, 32, 110–112). The correct conclusion is neither rejection nor acceptance. The correct conclusion is that syndrome-related constructs require better operationalization.

A cautious mapping can be proposed as a hypothesis-generating model. A Pi-Wei deficiency pattern may capture chronicity, poor appetite, fatigue, nutritional vulnerability, and impaired mucosal repair, and might be tested against advanced atrophy, low inflammatory activity, sarcopenia markers, or metabolomic depletion signatures. A Pi-Wei damp-heat pattern may capture activity, epigastric discomfort, sticky mouth sensation, bitter taste, red tongue or greasy coating, and could be tested against H. pylori activity, neutrophilic inflammation, erosive mucosa, cytokine markers, or dysbiotic profiles. Liver-Stomach disharmony may capture symptom reactivity, distension, belching, and neuroimmune or motility-related features rather than direct neoplastic transformation. Stomach-collateral blood stasis and phlegm-stasis interlocking may be tested against chronic field injury, angiogenic remodeling, hypoxia-related markers, or fibrotic stromal features. Stomach yin deficiency may be examined against mucosal atrophy, dryness-like symptom profiles, barrier compromise, and secretory dysfunction. None of these mappings should be written as equivalences; all should be written as testable latent-state hypotheses. These candidate syndrome-based host-response constructs and their validation hypotheses are summarized in **Table 3**.

**Table 3:** Candidate syndrome-based host-response constructs and validation hypotheses.

Syndrome construct	Possible host-response meaning	Candidate modern correlates	Evidence stance
Pi-Wei deficiency	Chronicity, poor appetite, fatigue, impaired repair, nutritional vulnerability	Atrophy, low-grade chronic injury, body-composition/nutritional markers, metabolomic depletion signatures	Hypothesis-generating; not lesion diagnostic
Pi-Wei damp-heat	Active inflammatory symptom cluster and mucosal irritation	H. pylori activity, neutrophilic inflammation, erosions, cytokine activation, dysbiosis, greasy tongue coating	Plausible field-host hypothesis; requires blinded testing
Liver-Stomach disharmony	Distension, belching, stress-related symptom reactivity, neuroimmune or motility component	Symptom burden, autonomic/motility markers, low correlation with focal pathology possible	Likely symptom-host phenotype rather than direct neoplasia marker
Stomach-collateral blood stasis	Chronic fixed pain, dark/purplish signs, possible microcirculatory emphasis	Irregular microvascular pattern, hypoxia/angiogenesis markers, stromal remodeling	Low-certainty hypothesis; avoid deterministic optical mapping
Stomach yin deficiency	Dryness-like symptom pattern, mucosal depletion, chronic atrophic state	Atrophy, barrier dysfunction, reduced secretory function, altered mucin expression	Hypothesis-generating
Phlegm-stasis interlocking	Longstanding complex host-field state with structural remodeling	Advanced premalignant field, fibrosis/stroma, immune-metabolic remodeling, dysplastic ecology	Exploratory; needs latent-cluster analysis

Digital tongue and tongue-coating studies are relevant because they begin to convert an historically subjective host-observable phenotype into quantifiable data. Machine-learning models based on tongue images have been developed for gastric precancerous lesion screening and gastric cancer diagnosis, and a multimodal pre-endoscopic risk model combining inquiry information and tongue images suggests a possible triage role. Metaproteomic analysis of tongue coating for gastric cancer risk screening offers a stronger molecular bridge between host-observable features and biological readouts. These studies are early, but they are more aligned with the present framework than treatment-only TCM reviews because they operationalize a host phenotype for prediction (18, 20, 88, 113, 114).

The methodological requirement is blinding. If the syndrome assessor knows the endoscopic or pathological diagnosis, the resulting syndrome assignment may be contaminated by expectation. If the endoscopist knows the syndrome label, lesion scoring may be biased. If the pathologist knows both, interpretation may be vulnerable to confirmatory pressure. A serious study must therefore separate syndrome assessment, endoscopic feature scoring, pathology review, and omics analysis, and must report inter-rater agreement for syndrome diagnosis. In that design, a negative result would be useful. If syndrome labels fail to add predictive value beyond age, sex, H. pylori status, OLGA/OLGIM stage, and endoscopic findings, the field will have learned something important.

Standardization should include both positive and negative controls. A syndrome form should be administered to patients with early gastric cancer, dysplasia, advanced intestinal metaplasia without visible neoplasia, non-atrophic gastritis, and functional dyspepsia-like symptoms when possible. This would help determine whether a syndrome category is truly related to neoplastic field biology or merely to upper gastrointestinal symptom burden. Without such controls, the study risks rediscovering symptom severity rather than cancer risk.

## 9 Toward an integrative host-response framework

The lesion-field-host framework is deliberately additive and falsifiable. Layer 1, the lesion phenotype, includes WLI, LCI/BLI, ME-NBI, Paris morphology, demarcation line, microvascular and microsurface patterns, biopsy result, ESD pathology, differentiation, invasion depth, ulceration, margin status, and lymphovascular invasion. Layer 2, the field phenotype, includes H. pylori status, eradication timing, atrophy, intestinal metaplasia distribution and subtype, SPEM-like metaplastic evidence where available, OLGA/OLGIM stage, Kyoto-classification features, microbiome profile, and field metabolomics. Layer 3, the host-response phenotype, includes age, sex, family history, nutritional status, inflammatory markers, symptoms, tongue or tongue-coating image features, structured syndrome label, and possibly plasma metabolomic or cytokine variables.

Temporal scale is an important feature of the lesion–field–host framework. Lesion-related variables are often event-defined and are anchored to the time of endoscopic detection, biopsy, or endoscopic resection. Field-related variables, such as glandular atrophy, intestinal metaplasia, SPEM-like metaplastic change, OLGA/OLGIM stage, and post-eradication mucosal remodeling, usually reflect long-term mucosal memory accumulated over years. In contrast, host-response variables, including symptoms, inflammatory activity, nutritional status, tongue-image features, and syndrome differentiation, may fluctuate over shorter periods. These layers should therefore not be interpreted as operating on the same biological timescale. Rather, a robust model should treat field variables as relatively slow-moving risk substrates and host-response variables as potentially dynamic modifiers or readouts that require date-stamped measurement, repeated assessment when feasible, and interpretation in relation to the timing of endoscopy, biopsy, eradication therapy, and treatment exposure.

The framework should be visualized as a hierarchy of evidence, not a hierarchy of importance. Lesion phenotype is decisive for immediate diagnosis and treatment. Field phenotype is decisive for background risk and surveillance. Host-response phenotype is exploratory for risk refinement and patient stratification. A study can fail at the host-response layer while still reinforcing lesion and field standards. This falsifiability is what distinguishes the framework from an interpretive essay.

The model is not meant to burden routine care with impractical tests. It is meant to guide research design and identify which variables are necessary to test a claim. For example, if a study claims that damp-heat syndrome is associated with early gastric neoplasia, it should specify whether the association is with focal lesion detection, background H. pylori activity, active neutrophilic inflammation, mucosal erosion, microbiome dysbiosis, cytokine profile, or patient symptoms. Without this specificity, a syndrome-neoplasia association is too broad to be interpretable.

The same discipline applies to AI. A model can be clinically useful only if the target is defined. Computer-aided detection of a suspicious lesion, computer-aided diagnosis of neoplasia, prediction of histologic differentiation, prediction of invasion depth, background risk classification, and host-state clustering are different tasks. Future models should follow TRIPOD+AI for reporting and PROBAST/PROBAST+AI for risk-of-bias assessment, with external validation, calibration, clinical-utility analysis, and transparent handling of center-level heterogeneity (115–117). If a systematic evidence map is developed as a supplement, PRISMA 2020 should guide reporting of search strategy and study selection (118).

**Figure 4:**
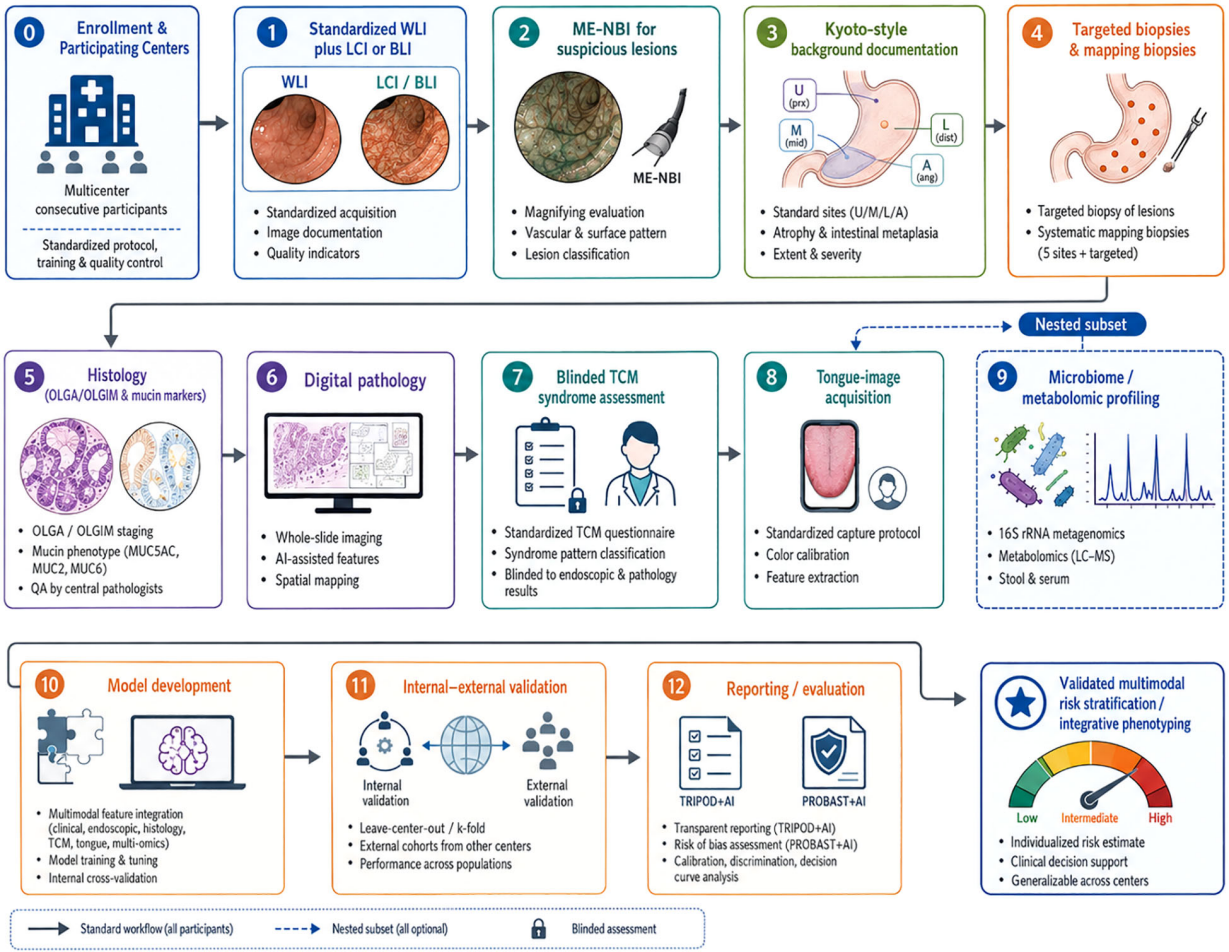
Proposed multicenter validation roadmap. Participants undergo standardized WLI plus LCI or BLI, ME-NBI for suspicious lesions, Kyoto-style background documentation, targeted and mapping biopsies, histology with OLGA/OLGIM and mucin markers, digital pathology, blinded TCM syndrome assessment, tongue-image acquisition, microbiome/metabolomic profiling in a nested subset, and model development with internal-external validation according to TRIPOD+AI and PROBAST+AI.

The most informative prospective design would be a multicenter cohort enrolling patients undergoing diagnostic upper endoscopy across screening, surveillance, and lesion-referral settings. A proposed multicenter validation roadmap for this lesion–field–host framework is shown in **Figure 4**. Each participant would undergo standardized WLI plus LCI or BLI, ME-NBI for suspicious lesions, background-mucosa photodocumentation, and recording of H. pylori status and eradication history. Biopsy and resection pathology would include updated Sydney variables, OLGA/OLGIM stage, dysplasia grade, histological type, invasion depth, mucin phenotype where available, and digital slide archiving. A blinded syndrome assessment would be performed before disclosure of endoscopic or pathological results, using a structured form and at least two trained assessors. A nested subset would include mucosal microbiome, plasma metabolomics, inflammatory markers, and multiplex immunohistochemistry or spatial profiling. Outcomes should be prespecified, such as high-grade dysplasia or early cancer, metachronous neoplasia, non-curative resection, or progression from advanced premalignant stages. The minimum dataset, standardization requirements, and bias-control measures for such studies are outlined in **Table 4**.

**Table 4:** Minimal dataset for future multicenter lesion-field-host studies.

Domain	Minimum required variables	Recommended standardization	Bias control
Clinical baseline	Age, sex, family history, smoking, alcohol, diet, medications, prior ESD, eradication history, symptoms	Uniform case-report form; prespecified risk groups	Avoid retrospective extraction-only designs where symptom and syndrome data are missing
Endoscopy	WLI, LCI/BLI where available, ME-NBI for suspicious lesions, lesion morphology, background mucosa, photo protocol	Standardized image set; central review; explicit detection vs characterization task	Endoscopist blinded to syndrome assessment; report interobserver agreement
Histology	Sydney variables, OLGA/OLGIM, dysplasia grade, cancer type, invasion depth, mucin phenotype, LVI, margins	Central pathology or double reading; digital slide archiving	Pathologists blinded to syndrome and AI labels
Host-response phenotype	Structured symptoms, tongue body/coating, pulse if used, syndrome label, nutritional and inflammatory markers	Two independent trained assessors; predefined diagnostic criteria	TCM assessors blinded to endoscopy/pathology; kappa reported
Omics subset	Mucosal microbiome, plasma/tissue metabolomics, cytokines, multiplex IHC/spatial profiling	Batch-controlled protocols; coded specimens; prespecified endpoints	Prevent data leakage; separate discovery and validation sets
Modeling	Conventional variables plus optical, histological, digital, omics, and host-response variables	TRIPOD+AI reporting; PROBAST/PROBAST+AI appraisal; external validation	Calibration, decision-curve analysis, missingness handling, center-level validation

Endpoint selection is critical. Detection of any early cancer, presence of high-grade dysplasia, progression of low-grade dysplasia, metachronous cancer after ESD, non-curative resection, and symptom improvement are not interchangeable outcomes. The proposed framework is most suited to oncologic and premalignant endpoints, not symptom relief alone. If a model is intended for surveillance, endpoints should include advanced OLGA/OLGIM stage, HGD/early cancer detection, and metachronous lesions. If it is intended for lesion characterization, endpoints should include histology, invasion depth, and curability after ESD.

A feasible alternative is a nested case-control study with early gastric cancer, dysplasia, and non-neoplastic atrophy or intestinal metaplasia controls matched by age, sex, and H. pylori status. Such a study would be less powerful for prediction, but it could test whether syndrome labels correlate primarily with field severity, optical lesion features, or systemic markers. A third option is a deeply phenotyped imaging-omics-pathology study in which latent clusters are generated from optical features, digital pathology, microbiome, metabolomics, and host-observable variables. This would be the most faithful test of the host-response hypothesis.

## 10 Clinical implications and limitations

Clinically, the lesion-field-host framework has three potential uses. First, it may improve surveillance logic. A small lesion in a low-risk field and a small lesion in a stomach with extensive incomplete intestinal metaplasia, severe corpus atrophy, persistent dysbiosis, and high-risk host markers should not be interpreted as the same clinical situation. Second, it may improve interpretation of post-eradication risk by combining eradication history with residual field injury rather than treating eradication as a binary cure. Third, it may create a disciplined pathway for integrative prevention, in which H. pylori eradication, endoscopic surveillance, lifestyle modification, nutritional support, symptom management, and evidence-based traditional medicine interventions are studied within the same risk framework rather than in parallel silos (6, 9, 58–60).

In practice, the safest near-term application is not to change treatment decisions for a diagnosed lesion. It is to improve pre-endoscopic triage, surveillance intensity, and research enrichment. For example, a patient with extensive atrophy, incomplete intestinal metaplasia, prior eradication, high-risk family history, and a reproducible host-response phenotype could be prioritized for high-quality image-enhanced surveillance. By contrast, no host-response category should reassure clinicians when endoscopy shows a suspicious lesion or when guideline-based surveillance is indicated.

The limitations are substantial. The strongest evidence in the review supports conventional endoscopy, histopathology, H. pylori eradication, OLGA/OLGIM staging, and management of premalignant conditions. The syndrome-differentiation evidence is weaker, more heterogeneous, and less directly tied to early gastric neoplasia. The review therefore does not claim that TCM syndrome differentiation can diagnose early cancer, predict invasion depth, or replace biopsy. It claims only that syndrome labels and related digital host-observable features may represent a candidate host-response layer worthy of standardized validation.

Reverse causation is a particular concern for host-response phenotyping. Several variables discussed in this review, including poor appetite, fatigue, nutritional vulnerability, symptom burden, tongue-coating changes, and syndrome labels, may arise as consequences of chronic mucosal injury, active inflammation, anxiety related to diagnosis, dietary restriction, or early neoplastic disease rather than as antecedent risk factors. Future studies should therefore distinguish pre-existing host vulnerability from disease-consequence markers. Methodologically, this requires prospective enrollment, syndrome and symptom assessment before disclosure of endoscopic or pathological results, adjustment for baseline atrophy, intestinal metaplasia, OLGA/OLGIM stage, H. pylori status, and lesion severity, and repeated measurements over time. Control groups with non-atrophic gastritis, advanced intestinal metaplasia without visible neoplasia, and functional dyspepsia-like symptoms may also help determine whether syndrome-related variables reflect neoplastic field biology or more general upper gastrointestinal symptom burden.

The review must also acknowledge geography. Much of the strongest endoscopic evidence comes from East Asian high-incidence settings with expert endoscopists and structured screening. Much of the syndrome literature comes from Chinese-language or integrative medicine contexts. Generalizing either literature to low-incidence regions or non-specialist endoscopy settings requires caution. This is another reason for multicenter validation and transparent reporting of center experience, disease prevalence, and endoscopist expertise.

Another limitation is that gastric carcinogenesis is not a single linear pathway. The Correa cascade is clinically useful but incomplete. Diffuse-type and poorly cohesive carcinomas, hereditary susceptibility, EBV-associated pathways, autoimmune gastritis, and H. pylori-negative contexts may not fit neatly into the intestinal metaplasia-centered sequence. A credible framework must preserve this complexity and avoid implying that every early cancer arises from the same field state (28, 69, 70).

Finally, integrative modeling risks overfitting. Adding endoscopy, pathology, microbiome, metabolomics, digital pathology, tongue-image data, and syndrome labels can generate apparently impressive clusters that fail externally. The proposed framework must therefore be validated with independent centers, predefined endpoints, adequate event counts, calibration, decision-curve analysis, and reporting of missingness. The aim is not to maximize variables, but to identify the smallest clinically useful set that improves risk stratification beyond established standards.

A practical safeguard is to begin with a conventional baseline model before adding novel layers. Age, sex, H. pylori status, eradication history, family history, atrophy, OLGIM stage, lesion morphology, and ME-NBI classification should be modeled first. Host-response and omics variables should then be tested for incremental value using prespecified metrics such as calibration, discrimination, reclassification, and decision-curve benefit. Without incremental-value analysis, a large multimodal model may appear sophisticated while offering no clinically meaningful advantage.

## 11 Conclusion

Early gastric neoplasia is not merely a small mucosal lesion waiting to be found. It is the visible and microscopic expression of a dynamic process that involves focal epithelial transformation, background mucosal injury, microbial and inflammatory ecology, metabolic adaptation, and host response. Modern endoscopy has refined lesion detection and characterization; histopathology defines tissue state and curability; molecular, single-cell, spatial, microbiome, and metabolomic tools are revealing the field biology behind visible lesions. The missing task is disciplined integration.

This conclusion is intentionally conservative. It does not ask readers to accept a new diagnostic doctrine. It asks them to accept that the visible lesion is only one layer of a larger process and that the next generation of studies should be designed accordingly. That is a modest but important shift: from cataloguing appearances to explaining why appearances emerge, where they emerge, and in which host-field context they are most clinically consequential.

The lesion-field-host framework proposed here offers a way to integrate these layers without confusing them. It protects the primacy of endoscopy and histopathology while allowing carefully measured host-response phenotypes, including syndrome differentiation and digital tongue or tongue-coating features, to be tested as supplementary variables. The highest-quality future studies will be prospective, multicenter, blinded, and multimodal. They will not ask whether a syndrome label is equivalent to a microscopic lesion. They will ask whether independently acquired host-response information improves the interpretation of lesion and field phenotypes, and whether that improvement changes surveillance, prevention, or clinical outcome.

## References

[1] Bray F Laversanne M Sung H Ferlay J Siegel RL Soerjomataram I . Global cancer statistics 2022: GLOBOCAN estimates of incidence and mortality worldwide for 36 cancers in 185 countries. CA Cancer J Clin. (2024) 74:229–63. doi: 10.3322/caac.21834 38572751

[2] Yao K Uedo N Kamada T Hirasawa T Nagahama T Yoshinaga S . Guidelines for endoscopic diagnosis of early gastric cancer. Dig Endosc. (2020) 32:663–98. doi: 10.1111/den.13684 32275342

[3] Japanese Gastric Cancer A . Japanese gastric cancer treatment guidelines 2021 (6th edition). Gastric Cancer. (2023) 26:1–25. doi: 10.1007/s10120-022-01331-8 36342574 PMC9813208

[4] Li P Li Z Linghu E Ji J Society of Digestive Endoscopy of the Chinese Medical Association CSGotCMACAoG Hepatologists NCRCfDDCMJCPGC . Chinese national clinical practice guidelines on the prevention, diagnosis, and treatment of early gastric cancer. Chin Med J (Engl). (2024) 137:887–908. doi: 10.1097/cm9.0000000000003101 38515297 PMC11046028

[5] Dinis-Ribeiro M Areia M de Vries AC Marcos-Pinto R Monteiro-Soares M O'Connor A . Management of precancerous conditions and lesions in the stomach (MAPS): guideline from the European Society of Gastrointestinal Endoscopy (ESGE), European Helicobacter Study Group (EHSG), European Society of Pathology (ESP), and the Sociedade Portuguesa de Endoscopia Digestiva (SPED). Virchows Arch. (2012) 460:19–46. doi: 10.1055/s-0031-1291491 22190006

[6] Pimentel-Nunes P Libanio D Marcos-Pinto R Areia M Leja M Esposito G . Management of epithelial precancerous conditions and lesions in the stomach (MAPS II): European Society of Gastrointestinal Endoscopy (ESGE), European Helicobacter and Microbiota Study Group (EHMSG), European Society of Pathology (ESP), and Sociedade Portuguesa de Endoscopia Digestiva (SPED) guideline update 2019. Endoscopy. (2019) 51:365–88. doi: 10.1055/a-0859-1883 30841008

[7] Dixon MF Genta RM Yardley JH Correa P . Classification and grading of gastritis. The updated Sydney System. International Workshop on the Histopathology of Gastritis, Houston 1994. Am J Surg Pathol. (1996) 20:1161–81. doi: 10.1097/00000478-199610000-00001 8827022

[8] Rugge M Meggio A Pennelli G Piscioli F Giacomelli L De Pretis G . Gastritis staging in clinical practice: the OLGA staging system. Gut. (2007) 56:631–6. doi: 10.1136/gut.2006.106666 17142647 PMC1942143

[9] Dinis-Ribeiro M Libanio D Uchima H Spaander MCW Bornschein J Matysiak-Budnik T . Management of epithelial precancerous conditions and early neoplasia of the stomach (MAPS III): European Society of Gastrointestinal Endoscopy (ESGE), European Helicobacter and Microbiota Study Group (EHMSG) and European Society of Pathology (ESP) Guideline update 2025. Endoscopy. (2025) 57:504–54. doi: 10.1055/a-2529-5025 40112834

[10] Zhang P Yang M Zhang Y Xiao S Lai X Tan A . Dissecting the single-cell transcriptome network underlying gastric premalignant lesions and early gastric cancer. Cell Rep. (2019) 27:1934–47:e5. doi: 10.1016/j.celrep.2019.04.052 31067475

[11] Kim J Park C Kim KH Kim EH Kim H Woo JK . Single-cell analysis of gastric pre-cancerous and cancer lesions reveals cell lineage diversity and intratumoral heterogeneity. NPJ Precis Oncol. (2022) 6:9. doi: 10.1038/s41698-022-00251-1 35087207 PMC8795238

[12] Wang R Song S Qin J Yoshimura K Peng F Chu Y . Evolution of immune and stromal cell states and ecotypes during gastric adenocarcinoma progression. Cancer Cell. (2023) 41:1407–26:e9. doi: 10.1016/j.ccell.2023.06.005 37419119 PMC10528152

[13] Huang KK Ma H Chong RHH Uchihara T Lian BSX Zhu F . Spatiotemporal genomic profiling of intestinal metaplasia reveals clonal dynamics of gastric cancer progression. Cancer Cell. (2023) 41:2019–37:e8. doi: 10.1016/j.ccell.2023.10.004 37890493 PMC10729843

[14] Zeng R Gou H Lau HCH Yu J . Stomach microbiota in gastric cancer development and clinical implications. Gut. (2024) 73:2062–73. doi: 10.1136/gutjnl-2024-332815 38886045 PMC11672014

[15] Sun DZ Xu L Wei PK Liu L He J . Syndrome differentiation in traditional Chinese medicine and E-cadherin/ICAM-1 gene protein expression in gastric carcinoma. World J Gastroenterol. (2007) 13:4321–7. doi: 10.3748/wjg.v13.i32.4321 17708604 PMC4250857

[16] Sun DZ Liu L Jiao JP Wei PK Jiang LD Xu L . Syndrome characteristics of traditional Chinese medicine: summary of a clinical survey in 767 patients with gastric cancer. Zhong Xi Yi Jie He Xue Bao. (2010) 8:332–40. doi: 10.3736/jcim20100406 20388473

[17] Zhang J Zhan Z Wu J Zhang C Yang Y Tong S . Association among lifestyle, clinical examination, polymorphisms in CDH1 gene and Traditional Chinese Medicine syndrome differentiation of gastric cancer. J Tradit Chin Med. (2013) 33:572–9. doi: 10.1016/s0254-6272(14)60023-6 24660577

[18] Ma C Zhang P Du S Li Y Li S . Construction of tongue image-based machine learning model for screening patients with gastric precancerous lesions. J Pers Med. (2023) 13. doi: 10.3390/jpm13020271 36836505 PMC9968136

[19] Wang L Zhang Q Zhang P Wu B Chen J Gong J . Development of an artificial intelligent model for pre-endoscopic screening of precancerous lesions in gastric cancer. Chin Med. (2024) 19:90. doi: 10.1186/s13020-024-00963-5 38951913 PMC11218324

[20] Chen J Sun Y Li J Lyu M Yuan L Sun J . In-depth metaproteomics analysis of tongue coating for gastric cancer: a multicenter diagnostic research study. Microbiome. (2024) 12:6. doi: 10.1186/s40168-023-01730-8 38191439 PMC10773145

[21] Yao K Doyama H Gotoda T Ishikawa H Nagahama T Yokoi C . Diagnostic performance and limitations of magnifying narrow-band imaging in screening endoscopy of early gastric cancer: a prospective multicenter feasibility study. Gastric Cancer. (2014) 17:669–79. doi: 10.1007/s10120-013-0332-0 24407989

[22] Zhang Q Wang F Chen ZY Wang Z Zhi FC Liu SD . Comparison of the diagnostic efficacy of white light endoscopy and magnifying endoscopy with narrow band imaging for early gastric cancer: a meta-analysis. Gastric Cancer. (2016) 19:543–52. doi: 10.1007/s10120-015-0500-5 25920526

[23] Hu YY Lian QW Lin ZH Zhong J Xue M Wang LJ . Diagnostic performance of magnifying narrow-band imaging for early gastric cancer: a meta-analysis. World J Gastroenterol. (2015) 21:7884–94. doi: 10.3748/wjg.v21.i25.7884 26167089 PMC4491976

[24] Rugge M Genta RM Fassan M Valentini E Coati I Guzzinati S . OLGA gastritis staging for the prediction of gastric cancer risk: a long-term follow-up study of 7436 patients. Am J Gastroenterol. (2018) 113:1621–8. doi: 10.1038/s41395-018-0353-8 30333540

[25] Yue H Shan L Bin L . The significance of OLGA and OLGIM staging systems in the risk assessment of gastric cancer: a systematic review and meta-analysis. Gastric Cancer. (2018) 21:579–87. doi: 10.1007/s10120-018-0812-3 29460004

[26] Toyoshima O Nishizawa T . Kyoto classification of gastritis: advances and future perspectives in endoscopic diagnosis of gastritis. World J Gastroenterol. (2022) 28:6078–89. doi: 10.3748/wjg.v28.i43.6078 36483157 PMC9724483

[27] Correa P . Human gastric carcinogenesis: a multistep and multifactorial process--First American Cancer Society Award Lecture on Cancer Epidemiology and Prevention. Cancer Res. (1992) 52:6735–40. doi: 10.1016/j.gtc.2013.01.002 1458460

[28] Correa P Piazuelo MB . The gastric precancerous cascade. J Dig Dis. (2012) 13:2–9. doi: 10.4172/2161-0681.1000147 22188910 PMC3404600

[29] Willet SG Lewis MA Miao ZF Liu D Radyk MD Cunningham RL . Regenerative proliferation of differentiated cells by mTORC1-dependent paligenosis. EMBO J. (2018) 37. doi: 10.15252/embj.201798311 29467218 PMC5881627

[30] Bockerstett KA Lewis SA Noto CN Ford EL Saenz JB Jackson NM . Single-cell transcriptional analyses identify lineage-specific epithelial responses to inflammation and metaplastic development in the gastric corpus. Gastroenterology. (2020) 159:2116–29:e4. doi: 10.1053/j.gastro.2020.08.027 32835664 PMC7725914

[31] Chen X Dai YK Zhang YZ Liu FB Lan SY Wang SS . Efficacy of traditional Chinese medicine for gastric precancerous lesion: a meta-analysis of randomized controlled trials. Complement Ther Clin Pract. (2020) 38:101075. doi: 10.1016/j.ctcp.2019.101075 31783342

[32] Zhong YL Wang PQ Hao DL Sui F Zhang FB Li B . Traditional Chinese medicine for transformation of gastric precancerous lesions to gastric cancer: a critical review. World J Gastrointest Oncol. (2023) 15:36–54. doi: 10.4251/wjgo.v15.i1.36 36684050 PMC9850768

[33] Correa P . A human model of gastric carcinogenesis. Cancer Res. (1988) 48:3554–60. 3288329

[34] Jencks DS Adam JD Borum ML Koh JM Stephen S Doman DB . Overview of current concepts in gastric intestinal metaplasia and gastric cancer. Gastroenterol Hepatol (N Y). (2018) 14:92–101. 29606921 PMC5866308

[35] Goldenring JR Mills JC . Cellular plasticity, reprogramming, and regeneration: metaplasia in the stomach and beyond. Gastroenterology. (2022) 162:415–30. doi: 10.1053/j.gastro.2021.10.036 34728185 PMC8792220

[36] Gonzalez CA Sanz-Anquela JM Gisbert JP Correa P . Utility of subtyping intestinal metaplasia as marker of gastric cancer risk. A review of the evidence. Int J Cancer. (2013) 133:1023–32. doi: 10.1002/ijc.28003 PMC3732516 23280711

[37] Li ML Hong XX Zhang WJ Liang YZ Cai TT Xu YF . Helicobacter pylori plays a key role in gastric adenocarcinoma induced by spasmolytic polypeptide-expressing metaplasia. World J Clin cases. (2023) 11:3714–24. doi: 10.12998/wjcc.v11.i16.3714 37383139 PMC10294147

[38] Chong Y Yu D Lu Z Nie F . Role and research progress of spasmolytic polypeptide-expressing metaplasia in gastric cancer (Review). Int J Oncol. (2024) 64. doi: 10.3892/ijo.2024.5621 38299264 PMC10836494

[39] Yao K . The endoscopic diagnosis of early gastric cancer. Ann Gastroenterol. (2013) 26:11–22. doi: 10.1007/978-981-10-6769-3_6 24714327 PMC3959505

[40] Muto M Yao K Kaise M Kato M Uedo N Yagi K . Magnifying endoscopy simple diagnostic algorithm for early gastric cancer (MESDA-G). Dig Endosc. (2016) 28:379–93. doi: 10.1111/den.12638 26896760

[41] He C Peng C Wang H Ouyang Y Zhu Z Shu X . The eradication of Helicobacter pylori restores rather than disturbs the gastrointestinal microbiota in asymptomatic young adults. Helicobacter. (2019) 24:e12590. doi: 10.1111/hel.12590 31124220

[42] Watanabe T Nadatani Y Suda W Higashimori A Otani K Fukunaga S . Long-term persistence of gastric dysbiosis after eradication of Helicobacter pylori in patients who underwent endoscopic submucosal dissection for early gastric cancer. Gastric Cancer. (2021) 24:710–20. doi: 10.1007/s10120-020-01141-w 33201352 PMC8065006

[43] Kitagawa Y Suzuki T Nankinzan R Ishigaki A Furukawa K Sugita O . Comparison of endoscopic visibility and miss rate for early gastric cancers after Helicobacter pylori eradication with white-light imaging versus linked color imaging. Dig Endosc. (2020) 32:769–77. doi: 10.1111/den.13585 31765047

[44] Shichijo S Uedo N Michida T . Detection of early gastric cancer after Helicobacter pylori eradication. Digestion. (2022) 103:54–61. doi: 10.1159/000519838 34727544

[45] Nakayoshi T Tajiri H Matsuda K Kaise M Ikegami M Sasaki H . Magnifying endoscopy combined with narrow band imaging system for early gastric cancer: correlation of vascular pattern with histopathology (including video). Endoscopy. (2004) 36:1080–4. doi: 10.1055/s-2004-825961 15578298

[46] Ezoe Y Muto M Uedo N Doyama H Yao K Oda I . Magnifying narrowband imaging is more accurate than conventional white-light imaging in diagnosis of gastric mucosal cancer. Gastroenterology. (2011) 141:2017–25:e3. doi: 10.1053/j.gastro.2011.08.007 21856268

[47] Kato M Kaise M Yonezawa J Toyoizumi H Yoshimura N Yoshida Y . Magnifying endoscopy with narrow-band imaging achieves superior accuracy in the differential diagnosis of superficial gastric lesions identified with white-light endoscopy: a prospective study. Gastrointest Endosc. (2010) 72:523–9. doi: 10.1016/j.gie.2010.04.041 20598685

[48] Li HY Dai J Xue HB Zhao YJ Chen XY Gao YJ . Application of magnifying endoscopy with narrow-band imaging in diagnosing gastric lesions: a prospective study. Gastrointest Endosc. (2012) 76:1124–32. doi: 10.1016/j.gie.2012.08.015 23025977

[49] Ok KS Kim GH Park Y Lee HJ Jeon HK Baek DH . Magnifying endoscopy with narrow band imaging of early gastric cancer: correlation with histopathology and mucin phenotype. Gut Liver. (2016) 10:532–41. doi: 10.5009/gnl15364 27021504 PMC4933412

[50] Dohi O Yagi N Majima A Horii Y Kitaichi T Onozawa Y . Diagnostic ability of magnifying endoscopy with blue laser imaging for early gastric cancer: a prospective study. Gastric Cancer. (2017) 20:297–303. doi: 10.1007/s10120-016-0620-6 27294430

[51] Zhao Y Dohi O Ishida T Yoshida N Ochiai T Mukai H . Linked color imaging with artificial intelligence improves the detection of early gastric cancer. Dig Dis. (2024) 42:503–11. doi: 10.1159/000540728 39102801

[52] Gao J Zhang X Meng Q Jin H Zhu Z Wang Z . Linked color imaging can improve detection rate of early gastric cancer in a high-risk population: a multi-center randomized controlled clinical trial. Dig Dis Sci. (2021) 66:1212–9. doi: 10.1007/s10620-020-06289-0 32363529

[53] Min M Sun X Bai J Zhang Q Yang X Guo Q . Diagnostic accuracy of linked colour imaging versus white light imaging for early gastric cancers: a prospective, multicentre, randomized controlled trial study. Ann Med. (2022) 54:3306–14. doi: 10.1080/07853890.2022.2147991 36411585 PMC9704855

[54] Yamaoka M Imaeda H Miyaguchi K Ashitani K Tsuzuki Y Ohgo H . Detection of early stage gastric cancers in screening laser endoscopy using linked color imaging for patients with atrophic gastritis. J Gastroenterol Hepatol. (2021) 36:1642–8. doi: 10.1111/jgh.15312 33125743

[55] Yoshifuku Y Sanomura Y Oka S Kurihara M Mizumoto T Miwata T . Evaluation of the visibility of early gastric cancer using linked color imaging and blue laser imaging. BMC Gastroenterol. (2017) 17:150. doi: 10.1186/s12876-017-0707-5 29216843 PMC5721593

[56] Fukuda H Miura Y Hayashi Y Takezawa T Ino Y Okada M . Linked color imaging technology facilitates early detection of flat gastric cancers. Clin J Gastroenterol. (2015) 8:385–9. doi: 10.1007/s12328-015-0612-9 26560036

[57] Fukuda H Miura Y Osawa H Takezawa T Ino Y Okada M . Linked color imaging can enhance recognition of early gastric cancer by high color contrast to surrounding gastric intestinal metaplasia. J Gastroenterol. (2019) 54:396–406. doi: 10.1007/s00535-018-1515-6 30291440

[58] Fukase K Kato M Kikuchi S Inoue K Uemura N Okamoto S . Effect of eradication of Helicobacter pylori on incidence of metachronous gastric carcinoma after endoscopic resection of early gastric cancer: an open-label, randomised controlled trial. Lancet. (2008) 372:392–7. doi: 10.1016/s0140-6736(08)61159-9 18675689

[59] Lee YC Chiang TH Chou CK Tu YK Liao WC Wu MS . Association between Helicobacter pylori eradication and gastric cancer incidence: a systematic review and meta-analysis. Gastroenterology. (2016) 150:1113–24. doi: 10.1053/j.gastro.2016.01.028 26836587

[60] Ford AC Yuan Y Moayyedi P . Helicobacter pylori eradication therapy to prevent gastric cancer: systematic review and meta-analysis. Gut. (2020) 69:2113–21. doi: 10.1016/s0016-5085(25)01052-2 32205420

[61] Ford AC Yuan Y Moayyedi P . Long-term impact of Helicobacter pylori eradication therapy on gastric cancer incidence and mortality in healthy infected individuals: a meta-analysis beyond 10 years of follow-up. Gastroenterology. (2022) 163:754–6. doi: 10.1053/j.gastro.2022.05.027 35598628

[62] Matsumura S Dohi O Yamada N Harusato A Yasuda T Yoshida T . Improved visibility of early gastric cancer after successful Helicobacter pylori eradication with image-enhanced endoscopy: a multi-institutional study using video clips. J Clin Med. (2021) 10. doi: 10.3390/jcm10163649 34441946 PMC8397151

[63] Hirasawa T Aoyama K Tanimoto T Ishihara S Shichijo S Ozawa T . Application of artificial intelligence using a convolutional neural network for detecting gastric cancer in endoscopic images. Gastric Cancer. (2018) 21:653–60. doi: 10.1007/s10120-018-0793-2 29335825

[64] Wu L He X Liu M Xie H An P Zhang J . Evaluation of the effects of an artificial intelligence system on endoscopy quality and preliminary testing of its performance in detecting early gastric cancer: a randomized controlled trial. Endoscopy. (2021) 53:1199–207. doi: 10.1055/a-1350-5583 33429441

[65] He X Wu L Dong Z Gong D Jiang X Zhang H . Real-time use of artificial intelligence for diagnosing early gastric cancer by magnifying image-enhanced endoscopy: a multicenter diagnostic study (with videos). Gastrointest Endosc. (2022) 95:671–8. doi: 10.1016/j.gie.2021.11.040 34896101

[66] Ling T Wu L Fu Y Xu Q An P Zhang J . A deep learning-based system for identifying differentiation status and delineating the margins of early gastric cancer in magnifying narrow-band imaging endoscopy. Endoscopy. (2021) 53:469–77. doi: 10.1055/a-1229-0920 32725617

[67] Iwaya M Hayashi Y Sakai Y Yoshizawa A Iwaya Y Uehara T . Artificial intelligence for evaluating the risk of gastric cancer: reliable detection and scoring of intestinal metaplasia with deep learning algorithms. Gastrointest Endosc. (2023) 98:925–33. doi: 10.1016/j.gie.2023.06.056 37392953

[68] Rugge M de Boni M Pennelli G de Bona M Giacomelli L Fassan M . Gastritis OLGA-staging and gastric cancer risk: a twelve-year clinico-pathological follow-up study. Aliment Pharmacol Ther. (2010) 31:1104–11. doi: 10.1111/j.1365-2036.2010.04277.x 20180784

[69] Solcia E Fiocca R Luinetti O Villani L Padovan L Calistri D . Intestinal and diffuse gastric cancers arise in a different background of Helicobacter pylori gastritis through different gene involvement. Am J Surg Pathol. (1996) 20:S8–S22. doi: 10.1097/00000478-199600001-00003 8694148

[70] Battista S Ambrosio MR Limarzi F Gallo G Saragoni L . Molecular alterations in gastric preneoplastic lesions and early gastric cancer. Int J Mol Sci. (2021) 22. doi: 10.3390/ijms22136652 34206291 PMC8268370

[71] Miao ZF Lewis MA Cho CJ Adkins-Threats M Park D Brown JW . A dedicated evolutionarily conserved molecular network licenses differentiated cells to return to the cell cycle. Dev Cell. (2020) 55:178–91. doi: 10.1016/j.devcel.2020.07.005 32768422 PMC7606764

[72] Miao ZF Sun JX Adkins-Threats M Pang MJ Zhao JH Wang X . DDIT4 licenses only healthy cells to proliferate during injury-induced metaplasia. Gastroenterology. (2021) 160:260–71. doi: 10.1053/j.gastro.2020.09.016 32956680 PMC7857017

[73] Saenz JB Vargas N Cho CJ Mills JC . Regulation of the double-stranded RNA response through ADAR1 licenses metaplastic reprogramming in gastric epithelium. JCI Insight. (2022) 7. doi: 10.1172/jci.insight.153511 PMC8855806 35132959

[74] Willet SG Thanintorn N McNeill H Huh SH Ornitz DM Huh WJ . SOX9 governs gastric mucous neck cell identity and is required for injury-induced metaplasia. Cell Mol Gastroenterol Hepatol. (2023) 16:325–39. doi: 10.1016/j.jcmgh.2023.05.009 37270061 PMC10444955

[75] Miao ZF Sun JX Huang XZ Bai S Pang MJ Li JY . Metaplastic regeneration in the mouse stomach requires a reactive oxygen species pathway. Dev Cell. (2024) 59:1175–91. doi: 10.1016/j.devcel.2024.03.002 38521055

[76] Liu KS Wong IO Leung WK . Helicobacter pylori associated gastric intestinal metaplasia: treatment and surveillance. World J Gastroenterol. (2016) 22:1311–20. doi: 10.3748/wjg.v22.i3.1311 PMC4716041 26811668

[77] Huang RJ Wichmann IA Su A Sathe A Shum MV Grimes SM . A spatially mapped gene expression signature for intestinal stem-like cells identifies high-risk precursors of gastric cancer. In: Biorxiv. Cold Spring Harbor, NY: Cold Spring Harbor Laboratory Press. (2023).

[78] Burclaff J Willet SG Saenz JB Mills JC . Proliferation and differentiation of gastric mucous neck and chief cells during homeostasis and injury-induced metaplasia. Gastroenterology. (2020) 158:598–609. doi: 10.1053/j.gastro.2019.09.037 31589873 PMC7010566

[79] Lee SH Jang B Min J Contreras-Panta EW Presentation KS Delgado AG . Up-regulation of Aquaporin 5 defines spasmolytic polypeptide-expressing metaplasia and progression to incomplete intestinal metaplasia. Cell Mol Gastroenterol Hepatol. (2022) 13:199–217. doi: 10.1016/j.jcmgh.2021.08.017 34455107 PMC8593616

[80] Parsonnet J Friedman GD Vandersteen DP Chang Y Vogelman JH Orentreich N . Helicobacter pylori infection and the risk of gastric carcinoma. N Engl J Med. (1991) 325:1127–31. doi: 10.1016/s0891-5520(05)70417-7 1891020

[81] Uemura N Okamoto S Yamamoto S . The aerosol society drug delivery to the lungs XI. London, United Kingdom, december 11-12, 2000. Abstracts. J Aerosol Med. (2001) 14:119–34. 10.1089/08942680152007981 11547709

[82] Moss SF . The clinical evidence linking Helicobacter pylori to gastric cancer. Cell Mol Gastroenterol Hepatol. (2017) 3:183–91. doi: 10.1016/j.jcmgh.2016.12.001 28275685 PMC5331857

[83] Wong BC Lam SK Wong WM Chen JS Zheng TT Feng RE . Helicobacter pylori eradication to prevent gastric cancer in a high-risk region of China: a randomized controlled trial. JAMA. (2004) 291:187–94. doi: 10.1001/jama.291.2.187 14722144

[84] Liou JM Malfertheiner P Lee YC Sheu BS Sugano K Cheng HC . Screening and eradication of Helicobacter pylori for gastric cancer prevention: the Taipei global consensus. Gut. (2020) 69:2093–112. doi: 10.1136/gutjnl-2020-322368 33004546

[85] Yan L . Effect of Helicobacter pylori eradication on gastric cancer incidence and mortality. (2022) 163:154–62. 10.1053/j.gastro.2022.03.039 35364066

[86] Lamb A Chen LF . Role of the Helicobacter pylori-induced inflammatory response in the development of gastric cancer. J Cell Biochem. (2013) 114:491–7. doi: 10.1002/jcb.24389 22961880 PMC3909030

[87] Wang A Nie S Lv Z Wen J Yuan Y . Infiltration of immunoinflammatory cells and related chemokine/interleukin expression in different gastric immune microenvironments. J Immunol Res. (2020) 2020:2450569. doi: 10.1155/2020/2450569 33426088 PMC7774301

[88] Wang ST Yang HW Zhang WL Li Z Ji R . Disruption of the gastric epithelial barrier in Correa's cascade: clinical evidence via confocal endomicroscopy. Helicobacter. (2024) 29:e13065. doi: 10.1111/hel.13065 38443332

[89] Gan S Li C Hou R Tian G Zhao Y Ren D . Dynamic changes of the immune microenvironment in the development of gastric cancer caused by inflammation. Mol Ther Oncol. (2024) 32:200849. doi: 10.1016/j.omton.2024.200849 39228396 PMC11369508

[90] Su Y Zhang X Liang Y Sun J Lu C Huang Z . Integrated analysis of single-cell RNA-seq and bulk RNA-seq to unravel the molecular mechanisms underlying the immune microenvironment in the development of intestinal-type gastric cancer. Biochim Biophys Acta Mol Basis Dis. (2024) 1870:166849. doi: 10.1016/j.bbadis.2023.166849 37591405

[91] Huang YK Wang M Sun Y Di Costanzo N Mitchell C Achuthan A . Macrophage spatial heterogeneity in gastric cancer defined by multiplex immunohistochemistry. Nat Commun. (2019) 10:3928. doi: 10.1038/s41467-019-11788-4 31477692 PMC6718690

[92] Park CH Lee AR Lee YR Eun CS Lee SK Han DS . Evaluation of gastric microbiome and metagenomic function in patients with intestinal metaplasia using 16S rRNA gene sequencing. Helicobacter. (2019) 24:e12547. doi: 10.1111/hel.12547 30440093 PMC6587566

[93] Park JY Seo H Kang CS Shin TS Kim JW Park JM . Dysbiotic change in gastric microbiome and its functional implication in gastric carcinogenesis. Sci Rep. (2022) 12:4285. doi: 10.1038/s41598-022-08288-9 35277583 PMC8917121

[94] Barra WF Sarquis DP Khayat AS Khayat BCM Demachki S Anaissi AKM . Gastric cancer microbiome. Pathobiology. (2021) 88:156–69. doi: 10.1159/000512833 33588422

[95] Yang J Zhou X Liu X Ling Z Ji F . Role of the gastric microbiome in gastric cancer: from carcinogenesis to treatment. Front Microbiol. (2021) 12:641322. doi: 10.3389/fmicb.2021.641322 33790881 PMC8005548

[96] Guo Y Cao XS Guo GY Zhou MG Yu B . Effect of Helicobacter pylori eradication on human gastric microbiota: a systematic review and meta-analysis. Front Cell Infect Microbiol. (2022) 12:899248. doi: 10.3389/fcimb.2022.899248 35601105 PMC9114356

[97] Kuligowski J Sanjuan-Herraez D Vazquez-Sanchez MA Brunet-Vega A Pericay C Ramirez-Lazaro MJ . Metabolomic analysis of gastric cancer progression within the Correa's cascade using ultraperformance liquid chromatography-mass spectrometry. J Proteome Res. (2016) 15:2729–38. doi: 10.1021/acs.jproteome.6b00281 27384260

[98] Huang S Guo Y Li ZW Shui G Tian H Li BW . Identification and validation of plasma metabolomic signatures in precancerous gastric lesions that progress to cancer. JAMA Netw Open. (2021) 4:e2114186. doi: 10.1001/jamanetworkopen.2021.14186 34156450 PMC8220475

[99] Li B Shu X Jiang H Shi C Qi L Zhu L . Plasma metabolome identifies potential biomarkers of gastric precancerous lesions and gastric cancer risk. Metabolomics. (2023) 19:73. doi: 10.1007/s11306-023-02037-3 37561286

[100] Choi SJ Choi HS Kim H Lee JM Kim SH Yoon JH . Gastric cancer and intestinal metaplasia: differential metabolic landscapes and new pathways to diagnosis. Int J Mol Sci. (2024) 25. doi: 10.3390/ijms25179509 39273456 PMC11395121

[101] Tian M Yao Z Zhou Y Gan Q Wang L Lu H . Deeprisk network: an AI-based tool for digital pathology signature and treatment responsiveness of gastric cancer using whole-slide images. J Transl Med. (2024) 22:182. doi: 10.1186/s12967-023-04838-5 38373959 PMC10877826

[102] Kobayashi M Takeuchi M Ajioka Y Hashimoto S Sato A Narisawa R . Mucin phenotype and narrow-band imaging with magnifying endoscopy for differentiated-type mucosal gastric cancer. J Gastroenterol. (2011) 46:1064–70. doi: 10.1007/s00535-011-0418-6 21667151

[103] Doyama H Yoshida N Tsuyama S Ota R Takeda Y Nakanishi H . The "white globe appearance" (WGA): a novel marker for a correct diagnosis of early gastric cancer by magnifying endoscopy with narrow-band imaging (M-NBI). Endosc Int Open. (2015) 3:E120–4. doi: 10.1055/s-0034-1391026 26135651 PMC4477017

[104] Enjoji M Kohjima M Ohtsu K Matsunaga K Murata Y Nakamuta M . Intracellular mechanisms underlying lipid accumulation (white opaque substance) in gastric epithelial neoplasms: a pilot study of expression profiles of lipid-metabolism-associated genes. J Gastroenterol Hepatol. (2016) 31:776–81. doi: 10.1111/jgh.13216 26513060

[105] Omura H Yoshida N Hayashi T Miwa K Takatori H Tsuji H . Interobserver agreement in detection of "white globe appearance" and the ability of educational lectures to improve the diagnosis of gastric lesions. Gastric Cancer. (2017) 20:620–8. doi: 10.1007/s10120-016-0676-3 27915451

[106] Cheng J Xia J Zhuang Q Xu X Wu X Wan X . A new exploration of white globe appearance (WGA) in ulcerative lesions. Z Gastroenterol. (2020) 58:754–60. doi: 10.1055/a-1200-2287 32785912

[107] Lee J Cha S Kim J Kim JJ Kim N Jae Gal SG . Ensemble deep learning model to predict lymphovascular invasion in gastric cancer. Cancers (Basel). (2024) 16. doi: 10.21203/rs.3.rs-2596637/v1 38275871 PMC10814827

[108] Huang W Wang X Zhong R Li Z Zhou K Lyu Q . Multimodal radiopathomics signature for prediction of response to immunotherapy-based combination therapy in gastric cancer using interpretable machine learning. Cancer Lett. (2025) 631:217930. doi: 10.1016/j.canlet.2025.217930 40675469

[109] Wang X Jiang Y Yang S Wang F Zhang X Wang W . Foundation model for predicting prognosis and adjuvant therapy benefit from digital pathology in GI cancers. J Clin Oncol. (2025) 43:3468–81. doi: 10.1200/jco-24-01501 40168636

[110] Xu W Li B Xu M Yang T Hao X . Traditional Chinese medicine for precancerous lesions of gastric cancer: a review. BioMed Pharmacother. (2022) 146:112542. doi: 10.1016/j.biopha.2021.112542 34929576

[111] Weng J Wu XF Shao P Liu XP Wang CX . Medicine for chronic atrophic gastritis: a systematic review, meta- and network pharmacology analysis. Ann Med. (2023) 55:2299352. doi: 10.1080/07853890.2023.2299352 38170849 PMC10769149

[112] Zhang T Zhang B Xu J Ren S Huang S Shi Z . Chinese herbal compound prescriptions combined with Chinese medicine powder based on traditional Chinese medicine syndrome differentiation for treatment of chronic atrophic gastritis with erosion: a multi-center, randomized, positive-controlled clinical trial. Chin Med. (2022) 17:142. doi: 10.1186/s13020-022-00692-7 36550503 PMC9773465

[113] Yuan L Yang L Zhang S Xu Z Qin J Shi Y . Development of a tongue image-based machine learning tool for the diagnosis of gastric cancer: a prospective multicentre clinical cohort study. EClinicalMedicine. (2023) 57:101834. doi: 10.1016/j.eclinm.2023.101834 36825238 PMC9941057

[114] Shang Z Du ZG Guan B Ji XY Chen LC Wang YJ . Correlation analysis between characteristics under gastroscope and image information of tongue in patients with chronic gastriti. J Tradit Chin Med. (2022) 42:102–7. 10.19852/j.cnki.jtcm.2022.01.006 PMC10164623 35322639

[115] Collins GS Moons KGM Dhiman P Riley RD Beam AL Van Calster B . TRIPOD+AI statement: updated guidance for reporting clinical prediction models that use regression or machine learning methods. BMJ. (2024) 385:e078378. doi: 10.1136/bmj-2023-078378 38626948 PMC11019967

[116] Wolff RF Moons KGM Riley RD Whiting PF Westwood M Collins GS . PROBAST: a tool to assess the risk of bias and applicability of prediction model studies. Ann Intern Med. (2019) 170:51–8. doi: 10.7326/m18-1376 30596875

[117] Moons KGM Damen JAA Kaul T Hooft L Andaur Navarro C Dhiman P . PROBAST+AI: an updated quality, risk of bias, and applicability assessment tool for prediction models using regression or artificial intelligence methods. BMJ. (2025) 388:e082505. doi: 10.1136/bmj-2024-082505 40127903 PMC11931409

[118] Page MJ McKenzie JE Bossuyt PM Boutron I Hoffmann TC Mulrow CD . The PRISMA 2020 statement: an updated guideline for reporting systematic reviews. BMJ. (2021) 372:n71. doi: 10.31222/osf.io/v7gm2_v1 33782057 PMC8005924

